# HuR up-regulates cell surface PD-L1 via stabilizing CMTM6 transcript in cancer

**DOI:** 10.1038/s41388-021-01689-6

**Published:** 2021-03-01

**Authors:** Yanbin Liu, Xingzhi Li, Hui Zhang, Mingming Zhang, Yanli Wei

**Affiliations:** 1 Institute of Immunology and Molecular Medicine, Jining Medical University, Jining, China; 2 BioBox Sciences, Inc, Guangzhou, China; 3 Department of urological surgery, Longgang District People’s Hospital of Shenzhen, Shenzhen, China; 4 Department of Biochemistry, Zhongshan School of Medicine, Sun Yat-sen University, Guangzhou, China

**Keywords:** Tumour immunology, Molecular biology

## Abstract

Despite the well-established role of CMTM6 in the stabilization of cell surface PD-L1 in cancer cells, the mechanisms underlying CMTM6 expression and regulation are still largely unknown. Here we unexpectedly find a strikingly positive correlation between CMTM6 and Hu-Antigen R (HuR) expression in most types of cancer. Mechanistically, we elucidate HuR stabilizes CMTM6 mRNA via direct association with AU-rich elements (AREs) in its 3′UTR and predominantly up-regulates CMTM6, which is readily abolished by HuR-specific inhibitor, MS-444. Phenotypically, we notice abundant cell surface PD-L1 in HuR-high cancer cells, which significantly inhibits immune activation of co-cultured T cells as indicated by IL-2 production. Treatment with MS-444 completely relieves immune suppression imposed by HuR-overexpression and further stimulates immune responses. Ectopic HuR accelerates allograft tumor progression in vivo, which is greatly compromised by simultaneous administration with MS-444. Our study uncovers a novel mechanism in control of CMTM6 and therefore PD-L1 expression, and suggests the potential of combining HuR inhibitor with PD-1/PD-L1 antibodies for cancer immunotherapy.

## Introduction

Immunotherapies targeting immune checkpoints, such as PD-L1, have achieved unprecedented clinical success in a number of human cancers [1], which highlights the importance of understanding the molecular mechanisms underlying PD-L1 regulation [2]. Recently, two groups coincidently identified CKLF-like MARVEL transmembrane domain containing protein 6 (CMTM6) as a critical factor controlling cell surface PD-L1 stability, albeit distinct mechanisms of action have been proposed through either endosome recycling [3] or inhibition of ubiquitination-mediated degradation [4]. The following investigation suggested association between CMTM6 and molecular/clinical characteristics of malignancy and prognostic value of CMTM6 in gliomas [5]. In lung cancer, Gao et al. showed CMTM6 correlated with PD-L1 expression, and predicted clinical response to PD-1 pathway blockade [6]. Notably, Chen et al. found shRNA-mediated depletion of CMTM6 down-regulated PD-L1 expression in SCC7 tongue squamous cancer cells, and consequently delayed allograft tumor growth and augmented CD8+ and CD4+ T-cell infiltration, which was accompanied by decrease of PD-1^+^, TIM-3^+^, VISTA^+^, LAG-3^+^, and B7-H3^+^ exhausted T cells [7]. This pioneering study presented proof-of-concept for CMTM6-targeted therapy against human cancer.

Hu-Antigen R (HuR) is encoded by and belongs to the embryonic lethal abnormal vision-like family which consists of HuR, HuB, HuC and HuD. Hu protein was first identified as endogenous antigen for autoantibody discovered in a paraneoplastic context of small cell lung cancer, which is subsequently designated as anti-Hu syndromes [8, 9]. HuR contains three RNA recognition motifs (RRMs), among which RRM1 and RRM2 play crucial roles in RNA binding, whereas RRM3 is involved in cooperative assembly of HuR oligomer on target transcripts [10]. The biological function of HuR as an mRNA stabilizer, potently binding to AU-rich elements (AREs) to antagonize degradation signals, was first described in 1998 [11]. Multiple evidences accumulate in support of critical involvements of HuR in human malignancies [12–18] and motivate intensive interests in exploitation of potent inhibitors for therapeutic purposes [19–21].

Here we unexpectedly uncovered a strikingly positive correlation between CMTM6 and HuR in almost all types of human cancer, and further identified canonical AREs in the 3′UTR region of CMTM6 mRNA. Notably, through predominant control of CMTM6 expression, HuR is consequently involved in the regulation of cell surface PD-L1 and tumor immune evasion. Due to the lack of CMTM6-targeting chemicals, HuR inhibitors therefore may serve as an alternative to indirectly suppress PD-L1 expression and synergistically circumvent tumor immune escape in combination with PD-1/PD-L1 antibodies.

## Results

### HuR positively regulates CMTM6

**Fig. 1:**
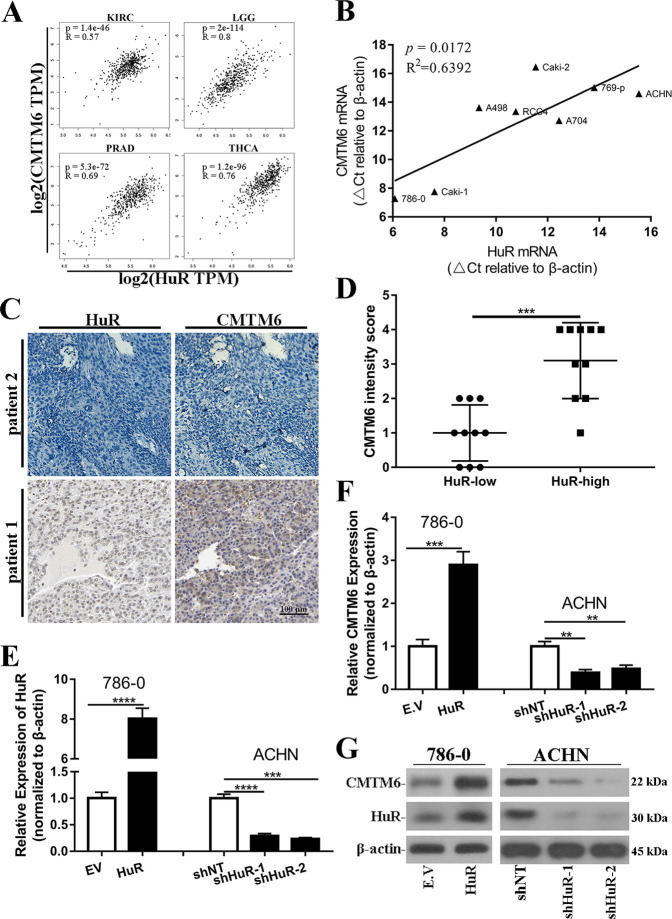
HuR positively regulated CMTM6. **A** Pearson correlation analysis of HuR and CMTM6 transcript in Kidney renal clear cell carcinoma (KIRC), Brain lower grade glioma (LGG), Prostate adenocarcinoma (PRAD) and Thyroid carcinoma (THCA) from TCGA database. **B** Correlation analysis of △Ct values of endogenous HuR and CMTM6 mRNAs in panel of ccRCC cell lines (786–0, 769-p, A498, A704, ACHN, Caki-1, Caki2, RCC4). **C** Representative images of IHC staining of HuR and CMTM6 protein in renal tumor samples. **D** Statistical comparison of CMTM6 intensity scores in HuR-low and -high renal tumors. **E** Establishment of HuR-overexpressing and -knockdown cell lines in 786–0 and ACHN cells, respectively. Relative expression of HuR was determined by real-time PCR. Three biological repeats (Mean ± SD). **F** Quantification of endogenous CMTM6 transcripts in 786–0 (E.V and HuR-overexpressing) and ACHN (control, shHuR-1 and shHuR-2) cells. Three biological repeats (Mean ± SD). **G** Western blots analysis of HuR and CMTM6 protein in 786–0 (E.V and HuR-overexpressing) and ACHN (control, shHuR-1 and shHuR-2) cells. * *p* < 0.05, ** *p* < 0.01, ***p < 0.001, ****p < 0.0001.

Despite the well-recognized role of CMTM6 in stabilizing cell surface PD-L1 [3, 4], the molecular mechanisms underlying CMTM6 regulation remain largely unknown. Here we performed correlation analysis using GEPIA2.0 (http://gepia2.cancer-pku.cn/) [22], and unexpectedly found a strikingly positive correlation between CMTM6 and HuR in a variety of human cancers (Fig. 1A and S1). The relevance was also noticed in a panel of clear cell renal cell carcinoma (ccRCC) cells (Fig. 1B). We further examined this at protein level with renal tumor tissues array, and IHC results clearly showed that CMTM6 was abundant in HuR-high samples (Fig. 1C, D). To clarify whether HuR was directly involved in CMTM6 regulation, we established stable cell lines with HuR-overexpression in 786–0 cells and HuR-knockdown in ACHN cells (Fig. 1E). Quantitative PCR results demonstrated that CMTM6 transcripts were significantly up-regulated in HuR-proficient 786–0 cells in comparison with empty vector control, while down-regulated in HuR-deficient ACHN cells compared to scrambled control (Fig. 1F). The positive regulation of CMTM6 by HuR was confirmed by immunoblotting as well (Fig. 1G). We additionally validated this phenotype in Caki-1 and 769-p cells (Fig. S2A-C). Therefore, our data suggested HuR as a positive regulator of CMTM6 in number of human cancers highly likely at transcript level. However, there were no significant differences in both HuR and CMTM6 expressions between normal and tumor tissues, which may suggest that HuR-CMTM6 is aberrantly high in a subpopulation of ccRCC (Fig. S3).

### Identification of AREs in 3′UTR of CMTM6

**Fig. 2:**
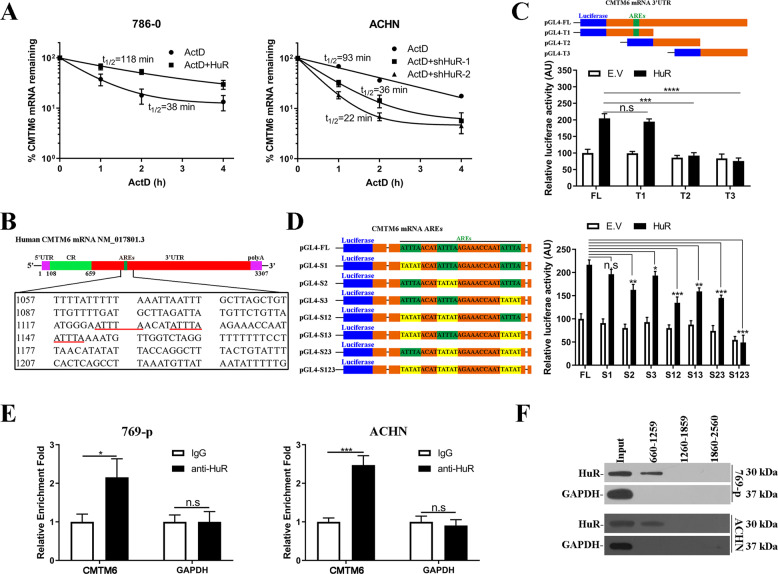
Identification of AREs in 3′UTR region of CMTM6 transcript. **A** CMTM6 mRNA decay in 786–0 (left) and ACHN (right) cells at 0, 1, 2, and 4 h post-treatment with 10 μg/ml of Actinomycin D. Three biological repeats. **B** Illustration of CMTM6 transcript with potential AREs sequences were underlined in red. **C** Relative luciferase activities were determined in full-length (FL) and truncated (T1, T2 and T3) CMTM6 3′UTR reporter plasmids while co-transfected with either empty control or HuR-overexpressing plasmids into 293T cells. Three biological repeats (Mean ± SD). **D** Scrambled mutants were generated in potential ARE elements either individual or in combination (left) in luciferase reporter plasmids, and relative luciferase activities were determined with co-transfection of either empty vector or HuR-overexpressing plasmids into 293T cells. Three biological repeats (Mean ± SD). **E** RNA-IP analysis of relative enrichment of CMTM6 transcripts in HuR-immunoprecipitate in both 769-p (left) and ACHN (right) cells. Three biological repeats (Mean ± SD). **F** Western blots examined HuR protein in pull-downed complex by indicated RNA probes in 769-p (upper) and ACHN (lower) cells. n.s: no significance, *p < 0.05, **p < 0.01, ***p < 0.001, ****p < 0.0001.

The physiological roles of HuR in stabilization of multiple mRNAs via direct binding to AREs were well-documented so far [16, 23–25], which prompted us to investigate the regulatory effects of HuR on CMTM6 along this direction. We first analyzed the stability of CMTM6 mRNA in response to HuR overexpression or knockdown. As shown in Fig. 2A, CMTM6 mRNA decay was greatly suppressed by ectopic introduction of HuR in 786–0, while expedited by HuR knockdown in ACHN cells, which underlined the stabilizing action of HuR on CMTM6 transcripts (Fig. S4). Next, we sought to identify the potential ARE motifs in 3′UTR of CMTM6 transcript. Close inspection of this region led to discovery of three putative AREs in tandem (Fig. 2B). We then constructed luciferase reporter plasmids fused to either full-length or serial truncations of CMTM6 3′UTR as illustrated in Fig. 2C. Co-transfection with HuR-expressing plasmids (Fig. S5) significantly stimulated luciferase activity of FL and T1 constructs rather than T2 and T3, which was in support of our prediction of AREs in T1 region. To further locate the HuR binding sites, we generated scrambled mutations of putative AREs either individually or in combination (Fig. 2D). One or two scrambled mutations introduced into AREs only partially compromised response to HuR overexpression, while all three mutations completely abolished the luciferase activity stimulated by HuR, which indicated the redundant and collaborative effects between three AREs sequences. The direct binding of HuR on CMTM6 3′UTR-fused luciferase mRNA and therefore half-life of luciferase transcript was evidently compromised by scramble mutations as well (Fig. S6). RNA-immunoprecipitation analysis demonstrated significant enrichment of CMTM6 transcripts in HuR-immunoprecipitated complex in both 769-p and ACHN cells (Fig. 2E), exhibiting the direct association between HuR and CMTM6 transcript. We further performed biotin-labelled RNA pulldown assay, and HuR was only detectable in the pulldown complex of RNA fragment 660–1259 nt (Fig. 2F). Taken together, we identified the canonical AREs in 3′UTR of CMTM6 mRNA and showed that CMTM6 is a direct target of HuR.

### HuR up-regulates cell surface PD-L1 via stabilization of CMTM6 transcript

**Fig. 3:**
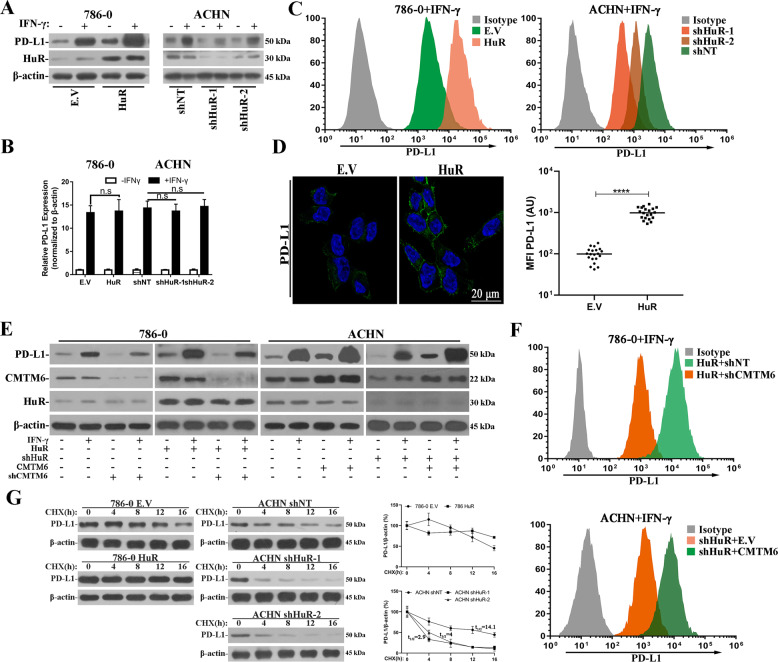
HuR up-regulated cell surface PD-L1 via stabilizing CMTM6. **A** Western blots analysis of PD-L1 in 786–0 (E.V and HuR-overexpressing) and ACHN (control, shHuR-1 and shHuR-2) cells without or with exposure to IFN-γ. **B** Quantitative PCR analysis of PD-L1 mRNA in 786–0 (E.V and HuR-overexpressing) and ACHN (control, shHuR-1 and shHuR-2) cells with or without exposure to IFN-γ. Three biological repeats (Mean ± SD). **C** Flow cytometry analysis of cell surface PD-L1 with PE-labelled anti-PD-L1 antibody in 786–0 (E.V and HuR-overexpressing, left) and ACHN (control, shHuR-1 and shHuR-2, right) cells. Representative of three experiments. **D** Immunofluorescence image of cell surface PD-L1 staining with anti-human PD-L1 antibody in 786–0 (E.V and HuR-overexpressing) cells (left). MFI, median fluorescence intensity (right). **E** Western blots analysis of PD-L1, CMTM6 and HuR protein in 786–0 (HuR-overexpressing in combination with either control or shCMTM6) and ACHN (shHuR-1 in combination with either empty control or HuR-overexpression) cells without or with exposure to IFN-γ. **F** Flow cytometry analysis of cell surface PD-L1 with PE-labelled anti-PD-L1 antibody in 786–0 (HuR-overexpressing in combination with either control or shCMTM6, upper) and ACHN (shHuR-1 in combination with either empty control or HuR-overexpression, lower) cells. Representative of three experiments. **G** Cells were dosed with 20 μM CHX and PD-L1 was analyzed by western blots at indicated time points. The intensity was estimated by densitometric scanning. PD-L1 protein stability represented three biological repeats (Mean ± SD). n.s: no significance.

Previous studies uncovered the critical roles of CMTM6 in stabilizing cell surface PD-L1, and consequently contributing to immune evasion of tumor cells. Our results suggested that CMTM6 transcript was subjected to stabilization by HuR binding, which prompted us to clarify the potential influences of HuR on cell surface PD-L1 abundance. Western blots analysis demonstrated IFN-γ-induced PD-L1 expression was remarkably increased by HuR-overexpression, and consistently decreased in HuR-deficient cells as well (Figs. 3A and S7A). The transcription and translation efficiency of PD-L1 remain constant regardless of HuR expression (Fig. 3B, S8). We further determined the cell surface PD-L1 using fluorescence labelled affinity antibody, and flow cytometry analysis showed decrease of PD-L1 abundance in HuR-deficient cells and remarkable increase in HuR-proficient cells (Fig. 3C). Similar results were observed with immunofluorescence staining in HuR-overexpressing 786–0 cells (Fig. 3D). We noticed a slightly but significantly positive correlation between HuR and PD-L1 protein in IHC analysis of renal tumors as well (Fig. S9).

To examine the predominant role of CMTM6 in mediating HuR-upregulated PD-L1, we next preformed rescue assay. Specific knockdown of CMTM6 in the context of HuR-overexpression significantly attenuated the IFN-γ-induced PD-L1 expression, while complementation with CMTM6 in HuR-deficient cells augmented IFN-γ-induced PD-L1 (Figs. 3E and S7B). Cell surface PD-L1 determined by fluorescence staining manifested evident decreases by CMTM6-knockdown in HuR-proficient cells and increase by CMTM6-overexpression in HuR-depleted cells (Fig. 3F). Consistent with previous reports, we noticed that ectopic HuR greatly suppressed the turnover rate of PD-L1 while HuR-deficiency significantly accelerated PD-L1 degradation in the presence of protein synthesis inhibitor cycloheximide (CHX, Fig. 3G). Our data unambiguously suggested that HuR up-regulated cell surface PD-L1 via stabilization of CMTM6 transcript. Both the co-localization and interaction of PD-L1 with CMTM6 were significantly stimulated by HuR-overexpression in 786–0 cells and partially compromised by HuR depletion in ACHN cells (Fig. S10).

### HuR inhibitor decreases cell surface PD-L1 via disruption of interaction between HuR and CMTM6

**Fig. 4:**
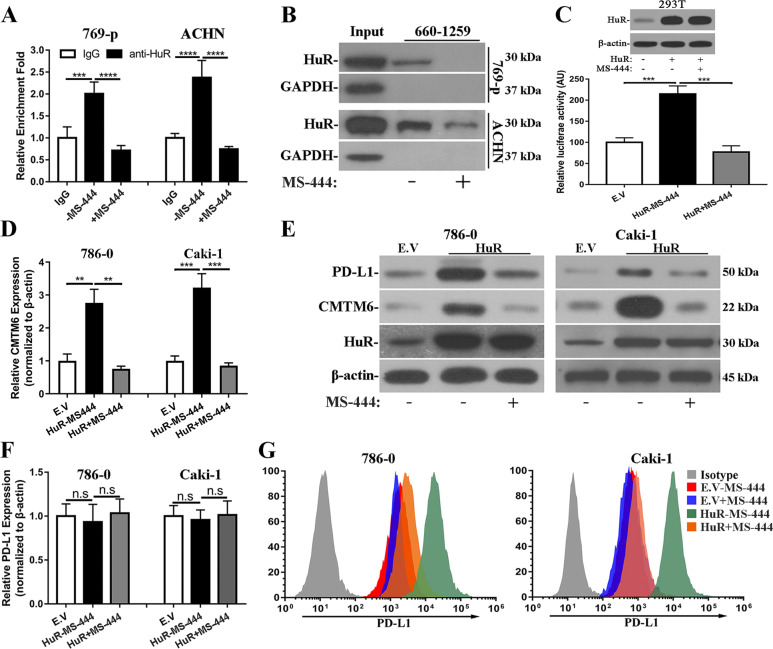
HuR inhibitor abolished PD-L1 up-regulation via disruption of interaction between HuR with CMTM6 mRNA. **A** RNA-IP analysis of CMTM6 transcript enrichment in presence of MS-444 in both 769-p (left) and ACHN (right) cells. Three biological repeats (Mean ± SD). **B** RNA pulldown analysis of association between HuR with indicated RNA probes in presence of MS-444 (50 μM) in 769-p (top) and ACHN (bottom). **C** Relative luciferase activities of pGL4-T1 in 293T (E.V and HuR-overexpressing) cells in presence of MS-444. Three biological repeats (Mean ± SD). **D** CMTM6 mRNA abundance was determined by real-time PCR in both 786–0 and Caki-1 (E.V and HuR-overexpressing) cells with or without MS-444 treatments. Three biological repeats (Mean ± SD). **E** Western blots analysis of PD-L1, CMTM6 and HuR proteins in 786–0 and Caki-1 (E.V and HuR-overexpressing) cells in presence of MS-444 (50 μM) and IFN-γ. **F** Quantitative PCR analysis of PD-L1 mRNA in 786–0 and Caki-1 (E.V and HuR-overexpressing) cells in presence of MS-444. Three biological repeats (Mean ± SD). **G** Flow cytometry analysis of cell surface PD-L1 in 786–0 and Caki-1 (E.V and HuR-overexpressing) cells in presence of MS-444. Representative of three experiments. n.s: no significance, ** *p* < 0.01, ***p < 0.001, ****p < 0.0001.

MS-444 is a cell permeable compound competitively binding to HuR and disrupting interaction between HuR and AREs of target mRNAs [19]. Here we employed MS-444 (dosing curve shown in Fig. S11) and found that treatment with MS-444 completely impaired enrichment of CMTM6 transcripts in HuR-immunoprecipitated complex in both 769-p and ACHN cells (Fig. 4A). In addition, HuR levels were significantly reduced in probe 660–1259-pulldowned protein species in the presence of MS-444 (Fig. 4B). We confirmed the constant expression of HuR at both transcript and protein levels under the conditions of MS-444 treatment (Fig. S12). The T1 luciferase reporter activities stimulated by HuR-overexpression were inhibited by co-treatment with MS-444 as well due to the disruption of association between HuR protein and luciferase mRNA (Figs. 4C and S13). Consequently, CMTM6 transcripts level up-regulated by HuR-overexpression was subsequently inhibited in the presence of MS-444 (Fig. 4D, half-life of CMTM6 mRNA was evaluated in Fig. S14). Western blots analysis demonstrated that PD-L1 was higher in HuR-proficient cells in response to IFN-γ exposure, which was tremendously down-regulated by MS-444 treatment (Fig. 4E). In line with the stabilizing effects of CMTM6 on PD-L1 protein, we did not observe notable changes in PD-L1 transcript levels upon HuR-overexpression alone or in combination with MS-444 treatments (Fig. 4F). Similar observations were noticed with another HuR-specific inhibitor, CMLD-2 (Fig. S15A–C). Flow cytometry analysis with fluorescence labelled affinity antibody displayed remarkable decreases in cell surface PD-L1 upon MS-444 exposure in both HuR-overexpressing 786–0 and Caki-1 cells (Fig. 4G). Our results showed that HuR inhibitor, MS-444, potently decreased cell surface PD-L1 through disruption of interaction between HuR and CMTM6 transcripts and downregulation of CMTM6.

### HuR inhibits immune activation

**Fig. 5:**
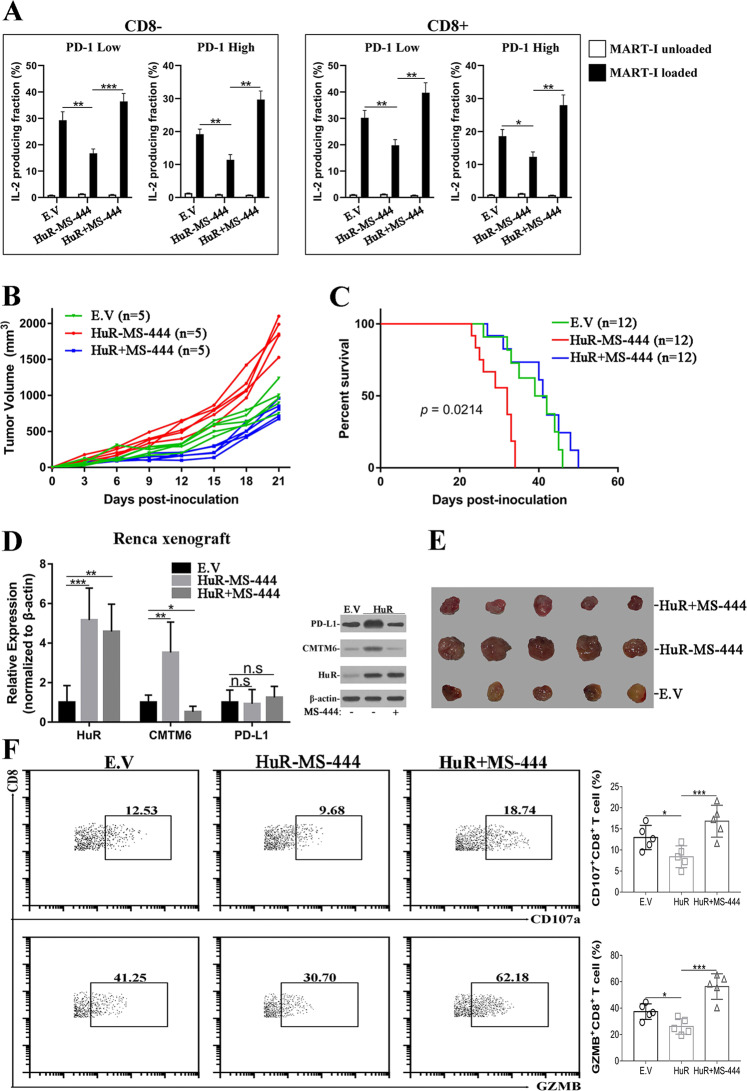
HuR inhibition enhanced immune activation in tumor. **A** IL-2 production by PD-1^Low^, PD-1^High^ primary human T cells transduced with the human MART-I-specific 1D3 TCR and PD-1, and co-cultured with MART-I peptide pre-loaded 786–0 cells (E.V and HuR-overexpressing) in absence or presence of MS-444 (50 μM). Representative of three experiments. **B** Renca allograft tumor (E.V, HuR-overexpressing alone or in combination with MS-444 treatment) growth curve. **C** Survival curve of Renca allograft tumor-bearing mice (E.V, HuR-overexpressing alone or in combination with MS-444 treatment). **D** Quantitative PCR examination of PD-L1, CMTM6 and HuR in allograft tumors derived from each group. Three biological repeats (Mean ± SD). **E** Macroscopic images of allograft tumors. **F** TILs were isolated from allograft tumors and analyzed by flow cytometry with anti-CD8, -CD107a and -GZMB immunofluorescent antibodies. Data are representative of five biological replicates. n.s: no significance, * *p* < 0.05, ** *p* < 0.01, ***p < 0.001.

The clinical success of PD-1-PD-L1-targeting therapeutics lies in potentiation of anti-tumor immunity. Our previous data showed that HuR up-regulated cell surface PD-L1 via stabilizing CMTM6, which made it a perfect therapeutic target to circumvent immune evasion. Here we employed cancer cell co-culture assays to evaluate the potential effects of HuR inhibitor on T cell response. IL-2 was measured which represents a key player in the cell-mediated immune response in allograft rejection and indicates local immune activation [26]. IL-2 secretion by Jurkat cells was significantly inhibited by HuR overexpression in 786–0 cells in comparison with empty vector control, while potentiated by HuR-knockdown in ACHN cells (Fig. S16A). The phenotype was completely reversed by PD-L1 depletion and overexpression, respectively. Simultaneous exposure to HuR inhibitor, MS-444, significantly restored IL-2 secretion which was compromised in HuR-overexpressed 786–0 and Caki-1 cells (Fig. S16B). In addition, human peripheral blood T cells were transduced with both MART-I ID8 T-cell receptor (TCR) and PD-1 (Low or High), and incubated with MART-I peptides pre-loaded parental or HuR-proficient 786–0 cells. We noticed relative reduction of IL-2 production by PD-1^High^ T cells upon addition of co-cultured tumor cells in comparison with PD-1^Low^, and HuR-proficiency in 786–0 cells significantly imposed suppressive effects on IL-2 production. Notably, treatment with HuR inhibitor, MS-444, completely abolished this suppression and further stimulated IL-2 production of T cells when compared with parental 786–0 cells (Fig. 5A). Consistent with previous report [3, 4], knockdown of either PD-L1 or CMTM6 greatly stimulated IL-2 production (Fig. S17). We examined effects of HuR on tumor control in vivo by employing HuR-overexpressed Renca allograft tumor model as well. Allograft tumor progression was significantly accelerated in HuR-high group compared to control mice, while greatly suppressed by simultaneous administration of MS-444 (Fig. 5B). The control and MS-444-treatment groups manifested comparable survival, which was much better than HuR-overexpressing mice (Fig. 5C). We validated the expression levels of HuR, CMTM6 and PD-L1 with real-time PCR and western blots in collected allograft tumor tissues (Fig. 5D). The representative macroscopic image of tumors from each group was provided in Fig. 5E. The cytotoxicity of tumor infiltrating CD8+ T cells was further determined by flow cytometry analysis. Both CD107a and GZMB subpopulation was significantly suppressed by HuR overexpression while tremendously increased in response to MS-444 (Fig. 5F). Our data uncovered the inhibitory effect of HuR on immune activation and highlighted the critical contributions of HuR to tumor progression in vivo via up-regulation of CMTM6-PD-L1 axis. Especially, we highlighted the potently therapeutic action of HuR inhibitor via re-activation of host immune response.

## Discussion

A comprehensive understanding of the molecular basis of PD-L1 regulation is crucial to the clinical success of PD-1/PD-L1-based immunotherapies. So far, a number of mechanisms underlying PD-L1 regulation in cancer have been uncovered, including gene copy number variation [27, 28], oncogenic signaling [29–31], exosomal transfer [32, 33], and microRNAs at posttranscriptional level [34, 35]. More recently, two elegant studies coincidently identified type-3 transmembrane protein CMTM6 as master factor in controlling cell surface PD-L1 stability [3, 4]. However, CMTM6 expression and regulation in cancer remain largely unknown. Here we found striking correlation between HuR and CMTM6 mRNA through bioinformatic analysis of publicly available TCGA database, which indicated a predominant role of HuR in control of CMTM6 transcript. The extremely short half-life of CMTM6 mRNA in the context of HuR-deficiency as noticed in mRNA decay assay suggested the intrinsic instability of this transcript, whose expression was heavily dependent on association with HuR protein. We further identified canonical HuR-recognizing sites in tandem in 3′UTR region of CMTM6 mRNA. Most importantly, via association with and stabilization of CMTM6, HuR was shown to upregulate cancer cell surface PD-L1 with exposure to IFN-γ, which was potently blockaded by HuR-specific inhibitor. HuR-overexpression greatly inhibited IL-2 secretion by T cells while HuR-knockdown stimulated IL-2 secretion in co-culture system, indicating a significantly inhibitory action of HuR on immune activation in vitro. Consistently, HuR-proficiency significantly accelerated Renca allograft tumor growth and associated with poorer survival, which was readily reversed by simultaneous administration of MS-444. Summarily, our study unraveled the importance of HuR in tumor immune evasion via regulating CMTM6-PD-L1 axis. Of note, our results cannot preclude the possibility that HuR was directly involved in PD-L1 regulation in view of the previously identified AREs in the 3′UTR of PD-L1 shown to be regulated by Tristetraprolin (TTP) [36, 37]. Furthermore, the potential role of TTP in competitively destabilizing CMTM6 needs to be investigated, especially in the cancers lacking correlation between CMTM6 and HuR. Although CMTM4 has also been shown to be involved in stabilizing PD-L1, our preliminary results demonstrated that CMTM4 is not directly regulated by HuR in ccRCC (Figs. S18 and 19).

The mRNA stabilizing effects of HuR have been well documented and multiple target genes have been identified, which were normally under regulation by complex networks. Notably, here we offered the first experimental evidence regarding HuR regulation of CMTM6 expression in cancer. And more importantly, the remarkable correlation between endogenous HuR and CMTM6 indicated the predominance of HuR in control of cellular CMTM6 transcripts notwithstanding other marginal mechanisms to be defined. The straightforward and stringent regulation of CMTM6 by HuR was demonstrated in our study in ccRCC cells, which might represent a universal mode-of-action in most types of cancer. We speculated that the closely clustered and appropriately dispersed multiple ARE motifs manifested the redundant and collaborative effects in its recognition by HuR and therefore were rendered significant stability to CMTM6 transcript.

Our data also supported the oncogenic properties of HuR as increasingly suggested by numerous investigations [18, 38, 39]. We showed that HuR-overexpression significantly increased cell surface PD-L1 via stabilizing CMTM6 and therefore compromised immune activation as indicated by IL-2 secretion in tumor cell-T cell co-culture system. In accordance with this finding, allograft tumors overexpressing HuR exhibited enhanced tumor growth compared to controls. Simultaneous administration with MS-444 completely abolished this pro-tumoral phenotype and reactivated local immune response, which critically underlined the importance of HuR in modulation of tumor immunity and implicated endogenous HuR as a valuable measure to predict the clinical response to PD-1-PD-L1-based immunotherapies, and more importantly, as an alternative target to CMTM6 for indirect interference of PD-L1 expression in tumor. Of note, the in vivo suppression of tumor growth by HuR inhibition also likely involved other pathways given the wide spectrum of oncogenic HuR targets.

As an essential oncogene with explicit mode-of-action, HuR attracted intensive interests for discovery and development of small-molecular chemical inhibitors. MS-444 and CMLD-2 were two prominent candidates to potently impair association between HuR with target AREs motif [19–21]. The following investigations have uncovered anti-cancer activities of these compounds and proposed distinct molecular events against multiple cancers. Wang et al. demonstrated that MS-444 treatment resulted in loss of cell viability and induction of apoptosis in glioblastoma cells accompanied by attenuation of mRNAs in some pro-tumoral pathways including angiogenesis, immune evasion and anti-apoptosis [40]. Intraperitoneal administration with MS-444 significantly diminished the number of small intestinal tumors generated in APC^Min^ familial adenomatosis polyposis and colon cancer model mice [41]. Blanco et al. reported that intraperitoneal administration of MS-444 was well-tolerated and suppressed xenograft CRC tumor progression through induced apoptosis and inhibited angiogenesis [42]. Also, MS-444 has been shown to exhibit anti-tumor effects against thyroid cancer cells via downregulation of MAD2 [43], and cytotoxicity toward human lung cancer cells via activation of cell cycle arrest and apoptosis [44]. In our study, immune cell secreted IL-2 was attenuated by HuR-overexpression in ccRCC cells and dramatically stimulated by MS-444 treatment, indicating an unrecognized role of HuR inhibitors in augmenting the immune response through downregulation of CMTM6-PD-L1. Our study strongly warrants further investigations into therapeutic potency of combination of PD-1/PD-L1 antibodies with HuR inhibitor.

## Materials and methods

### Cell culture

Human embryonic kidney cell line 293T, human ccRCC cell lines 786–0, 769-p, A498, A704, ACHN, Caki-1, Caki2, T lymphocyte cell Jurkat and murine renal carcinoma cell line Renca were obtained from ATCC (NY, USA). RCC4 cell was purchased from Sigma-Aldrich (MO, USA). 293T cells were maintained in DMEM high glucose and all other cancer cells were maintained in RPMI-1640 medium containing 10% FBS, 1% penicillin/streptomycin (Gibco, MA, USA). Cells were authenticated by STR profiling and tested as mycoplasma contamination-free before use. Transfection was performed using Lipofectamine 2000 (Invitrogen, MA, USA) following the manufacturer’s instructions.

### Plasmid constructs

The shHuR-1: GAGAACGAATTTGATCGTCAA, shHuR-2: GCAGCATTGGTGAAGTTGAAT; shCMTM6–1: GCTGCAATTGTGTTTGGATTT, shCMTM6–2: CTTTCTTCTGAGTCTCCTTAT; shPD-L1–1: CTGACATTCATCTTCCGTTTA, shPD-L1–2: GGCATTTGCTGAACGCATTTA were synthesized and cloned into pLKO.1 vector (Addgene, MA, USA) using T4 DNA ligase (New England Biolabs, MA, USA). The shGFP sequence ACAACAGCCACAACGTCTATA was cloned into pLKO.1 was used as scramble control. HuR (NM_001419), CMTM6 (NM_017801) and PD-L1 (NM_014143) was amplified by PCR and cloned into pcDNA3.1 vector for ectopic expressing purpose.

### Real-time PCR

Total RNA was collected from indicated cells and allograft tumors using TRIzol in accordance with the manufacturer’s manual (Invitrogen, MA, USA). Reverse transcription was performed with High-Capacity cDNA Reverse Transcription Kit (Thermo Fisher, MA, USA). Quantitative PCR was conducted with PowerUp SYBR Green Master Mix (Thermo Fisher) on CFX96 Touch PCR Detection System (Bio-Rad, CA, USA). β-actin was adopted as internal reference and relative abundance was determined by 2^-△△Ct^ method. Primer sequences were listed as follows:

HuR forward 5′-AACTACGTGACCGCGAAGG-3′, reverse 5′-CGCCCAAACCGAGAGAACA-3′; CMTM6 forward 5′-ATGAAGGCCAGCAGAGACAG-3′, reverse 5′-GTGTACAGCCCCACTACGGA-3′; PD-L1 forward 5′-GCTGCACTAATTGTCTATTGGGA-3′, reverse 5′-AATTCGCTTGTAGTCGGCACC-3′; GAPDH forward 5′-GGAGCGAGATCCCTCCAAAAT-3′, reverse 5′-GGCTGTTGTCATACTTCTCATGG-3′; β-actin forward 5′-CATGTACGTTGCTATCCAGGC-3′, reverse 5′-CTCCTTAATGTCACGCACGAT-3′.

### Immunohistochemistry

Human renal tumor tissue array was purchased from AlenaBio (Xi’an, China) and immunohistochemical staining was performed with Biotin-Streptavidin HRP Detection Systems (ZSGB-BIO, Beijing, China). Briefly, tissue array was first heated at 60 °C for 30 min, deparaffinated in xylene and rehydrated with gradient ethanol solution. Antigen was retrieved by boiling in sodium citrated solution (0.01 M, pH 6.0, 98 °C for 15 min). Endogenous peroxidase blocking was performed with 3% H_2_O_2_/methanol at room temperature for 10 min and followed by blocking with 10% FBS. Tissue assay was probed with primary antibodies (rabbit anti-HuR, HPA046298; rabbit anti-CMTM6, HPA026980, Sigma-Aldrich; rabbit anti-PD-L1, #13684, Cell Signaling Technology, MA, USA) on orbital shaker at 4 °C overnight, and then incubated with biotin-labelled secondary antibody at room temperature for 15 min. The HRP-streptavidin conjugates were applied for another 15 min at room temperature, and slides were detected with diaminobenzidine and counterstained with hematoxylin. Images were captured under DMi8 Inverted Microscope (Leica, Wetzlar, Germany).

### Western blots and immunoprecipitation

Cell lysates were prepared in ice-cold RIPA buffer with proteinase/phosphatase inhibitor cocktails (Roche, Basel, Switzerland). Protein concentration was determined with BCA Protein Assay Kit (Thermo Fisher). The protein species were resolved by 10% SDS-PAGE and transferred onto PVDF membrane (Millipore, MA, USA), which was then briefly blocked with 5% non-fat milk. Primary antibody (mouse anti-PD-L1, UMAB228, Origene, MD, USA; rabbit anti-CMTM6, HPA026980, Sigma-Aldrich; rabbit anti-HuR, #12582; rabbit anti-β-actin, #4967; rabbit anti-GAPDH, #2118; Cell Signaling Technology) hybridization was performed on orbital shaker at 4 °C overnight, and followed by 1 h of incubation with HRP-conjugated secondary antibodies (goat anti-rabbit, #7074; horse anti-mouse, #7076; Cell Signaling Technology) at room temperature for 1 h. Blots were visualized using ECL (Millipore). To determine the half-life of PD-L1, the indicated cells were treated with CHX (20 μM, Sigma) for up to 16 h and harvested for western blots analysis. Co-immunoprecipitation was performed with 500 μg of total cell lysates incubating with 20 μl of anti-PD-L1 antibody (rabbit anti-PD-L1, 13684, Cell Signalling Technology) at 4 °C overnight, followed by addition of protein protein G dynabeads and incubation for another 2 h at 4 °C. The immunoprecipitated complex was washed and eluted with SDS sample buffer for western blots analysis. All western blots were repeated in triplicate and representative images were presented.

### CMTM6 mRNA decay assay

786–0 (E.V and HuR-overexpressing) and ACHN (control, shHuR-1 and shHuR-2) cells were seeded into 6-well plate (3 × 10^5^ cells/well) and allowed for attachment overnight. Actinomycin D (10 μg/ml, Sigma-Aldrich) or α-amanitin (50 mM, Sigma-Aldrich) was added dropwise to each well and incubated for indicated time. RNA was then extracted as previously described and remaining transcript abundance was determined by real-time PCR.

### Luciferase assays

3′UTR region of CMTM6 transcript NM_017801.3 was amplified by PCR and cloned into pGL4 luciferase reporter vector (Promega, WI, USA) using Xba I site. PCR splicing method was adopted to generate truncate mutations, and site-directed mutagenesis PCR was used to generate scrambled mutations, respectively. CMTM6 3′UTR reporter (wild type or mutations) plasmids were co-transfected with either empty control or HuR-overexpressing plasmids into 293T cells for 48 h. Cells were collected for relative luciferase activity measurement with Bright-Glo Luciferase Assay System (Promega) according to the manufacturer’s instruction on microplate reader (BioTek, VT, USA). Luciferase mRNA decay in response to mutations introduced into AREs motif of CMTM6 3′UTR was determined as previously described.

### RNA-immunoprecipitation

RNA-immunoprecipitation assay was performed with Magna RIP RNA-Binding Protein Immunoprecipitation Kit (Millipore). The exponential cells were washed with ice-cold PBS and collected by centrifugation into complete RIP lysis buffer on ice for 5 min and followed by immediate freezing at −80 °C. The primary antibodies (anti-HuR, #12582, Cell Signaling Technology) were pre-bound to protein A/G magnetic beads on rotator at room temperature for 30 min, and then incubated with cell lysates overnight at 4 °C. After rigorous wash, the immunoprecipitated complexes were digested with proteinase K at 55 °C for 30 min. RNA species were recovered using TRIzol reagent and cDNA was prepared as previously described. Enriched HuR and GAPDH transcripts were analyzed by real-time PCR.

### RNA pulldown assay

RNA pulldown assay was performed with Pierce Magnetic RNA-Protein PullDown Kit (Thermo Fisher) following the manufacturer’s instructions. Based on luciferase reporter results, we designed three sequential RNA probes across 660–1259, 1260–1859 and 1860–2560 of CMTM6 transcript, respectively. RNA probes were obtained from corresponding cDNA fragments using MAXIscript SP6 Transcription Kit (Thermo Fisher). Pierce RNA 3′ End Desthiobiotinylation Kit was used to label RNA probes, and 50 pmol of labelled RNA was immobilized on 50 μl of streptavidin magnetic beads for 30 min at room temperature with agitation. Cell lysates were prepared in IP Lysis buffer and 50 μg of cell lysates were incubated with RNA probes for 1 h at 4 °C on rotator. Protein species were recovered by elution and analyzed by immunoblotting.

### Flow cytometry

The indicated cells were pretreated with 500 IU/ml IFN-γ (Peprotech, NJ, USA) for 48 h and collected in PBS by trypsin digestion. After wash three times, cell pellets were resuspended in 2% BSA and 100 μl aliquot (1 × 10^6^ cells) was used for antibody labelling with PE-anti-PD-L1 antibody (329705, BioLegend, CA, USA) at 4 °C for 15 min in the dark. After wash with staining buffer twice and filtered with cell strainer, the single-cell suspension was prepared on ice for flow cytometry analysis on Gallios (Beckman Coulter, CA, USA).

### Immunofluorescence

HuR-overexpressing and control 786–0 cells were plated on cover slips and cultured for 24 h. Cells were fixed by 4% PFA for 15 min, permeabilized by 0.25% Triton X-100 for 15 min and blocked with 5% BSA/PBS at room temperature for 1 h. Primary antibody (goat anti-PD-L1, PA5–18337, Thermo Fisher) was then incubated in humidified chamber at room temperature for 1 h, followed by rigorous wash with PBST. Alexa Fluor 488-labelled secondary antibody (donkey anti-goat, A32814, Thermo Fisher) was incubated for another hour in the dark. Immediately after PBST wash, the cover slips were mounted with ProLong^™^ Gold Antifade Mountant with DAPI (Sigma-Aldrich). The images were captured under confocal microscope (LSM800, Carl Zeiss, Oberkochen, Germany).

### Polysome profiling

Cell lysate from 786–0 cells (E.V and HuR) was prepared after 10 min incubation with CHX (100 μg/ml) in order to inhibit ribosomal translocation and freeze polysome on mRNA. After centrifugation at 12,000 *g* for 15 min (4 °C), all cell debris were discarded and the supernatant was carefully layered onto a 10–50% linear sucrose gradient and centrifuged at 39,000 *g* for 3 h at 4 °C. Fractions were collected and absorbance at 254 nm was monitored. The relative abundance of both PD-L1 and β-actin transcripts were determined by real-time PCR.

### Jurkat co-culture IL-2 secretion

786–0 (E.V and HuR-overexpressing) and ACHN (control, shHuR-1 and shHuR-2) cells were subjected to pre-treatment with IFN-γ (500 IU/ml) for 24 h. Jurkat cells were pre-activated with PMA (25 ng/ml, P1585, Sigma-Aldrich) and PHA (1 μg/ml, L2769, Sigma-Aldrich) for 24 h. Co-culture was performed at the ratio of 2:1 Jurkat: 786–0/ACHN cells. The secreted IL-2 in culture medium was quantified with IL-2 Human ELISA Kit (Invitrogen) at 48 h and 72 h, respectively. For MS-444 (Sigma-Aldrich) dosage, 50 μM of MS-444 was added at the beginning of co-culture.

### IL-2 production assay

Human peripheral blood T cells were obtained from STEMCELL (Vancouver, Canada) and transduced with both MART-I-specific 1D3 T cell receptor (TCR) and PD-1. 786–0 (E.V and HuR-overexpression) cells were pre-loaded with MART-I peptides (10 ng/ml) at 37 °C for 1 h, and incubated with indicated T cells at a ratio of 1:1 in the presence of protein transport inhibitor Golgiplug (1 μl/ml, BD Biosciences, CA, USA). After 5 h incubation, cells were washed and stained with FITC-labelled anti-CD8 (MCD0801, Thermo Fisher, MA, USA), and the intracellular IL-2 production was determined by flow cytometry with APC-labelled anti-Human IL-2 (554567, BD Biosciences, CA, USA).

### Analysis of tumor infiltrating lymphocytes (TILs)

Allograft tumors were collected and gently minced, and followed by enzymatic digestion (200 μg/ml of collagenase IV and 50 μg/ml of DNase I in PBS) at 37 °C for 1 h with rotation. The resultant mixture was filtered with 70 μm cell strainer and cell pellets were collected by centrifugation at 2000 *rpm* for 5 min. TILs were further enriched using Percoll gradient (17089102, Cytiva, MA, USA) following the manufacturer’s instruction, and CD107a (APC labelled, sab4700417, Sigma, MO, USA) and GZMB (FITC-labelled, sab4700295, Sigma, MO, USA) subpopulation was analyzed by flow cytometry as previously described.

### Renca allograft animal model

Mice study was approved by the Institutional Animal Care and Use Committee (IACUC) of Jining Medical University. The BALB/c mice (4-week-old) were purchased from Vital River Laboratory (Beijing, China) and quarantined/acclimated for 1 week. Renca stable cells (2 × 10^6^ E.V or HuR-overexpression) were homogenously mixed with equal volume of Matrigel (Corning, NY, USA) and s.c. inoculated into lower flank of mice. Tumor growth was estimated by digital caliper and tumor volume was calculated as length × width^2^ × 0.5. The BALB/c mice were intraperitoneally injected with MS-444 every 3 days (10 mg/kg prepared in 10% N-methyl-2-pyrrolidone in PBS) once tumor volumes reached 50 mm^3^.

### Statistical analysis

Data processing and analysis were performed with GraphPad Prism 7.0. The unpaired, two-tailed Student’s *t* test was employed for statistical comparison, and *p* < 0.05 was considered as significant difference.

## Supplementary information

supplementary figure legends

Figure S1. RNA expression of HuR in human cancers and correlates with CMTM6 mRNA levels.

Figure S2. HuR up-regulated CMTM6 in Caki-1 and 769-p cells.

Figure S3. Relative expression of HuR and CMTM6 mRNA in KIRC.

Figure S4. CMTM6 mRNA decay in Caki-1 (E.V and HuR-overexpressing, left) and 769-p (shNT, shHuR-1 and shHuR-2, right) cells.

Figure S5. Overexpression of HuR in 293T.

Figure S6. Scramble mutations of AREs disrupted binding of HuR to CMTM6 3’UTR-fused luciferase and compromised its stability.

Figure S7. HuR up-regulated cell surface PD-L1 via CMTM6.

Figure S8. HuR imposed no significant influences on PD-L1 translation efficiency.

Figure S9. Correlation analysis of HuR and PD-L1 in renal tumors based on IHC intensity scores.

Figure S10. Co-localization and interaction of PD-L1 with CMTM6 in response to HuR overexpression and knockdown.

Figure S11. MS-444 decreased HuR-upregulatedCMTM6 transcript levels.

Figure S12. MS-444 showed no influences on HuR expression.

Figure S13. MS-444 abolished binding of HuR on CMTM6 3’UTR-fused luciferase.

Figure S14. Prolonged half-life of CMTM6 transcripts in HuR-proficient cells was decreased by MS-444 treatment.

Figure S15. HuR inhibition with CMLD-2 abolished both CMTM6 and PD-L1 up-regulation.

Figure S16. MS-444 restored IL-2 secretion suppressed by HuR in 786–0 and Caki-1 cells.

Figure S17. The impacts of HuR-, CMTM6- and PD-L1 knockdown on IL-2 production.

Figure S18. Correlation analysis of HuR with CMTM4 mRNA levels in TCGA human cancers.

Figure S19. HuR showed no significant regulation on CMTM4 expression.
