# Supplementary material for: HuR up-regulates cell surface PD-L1 via stabilizing CMTM6 transcript in cancer
Source: Oncogene. 2021 Mar 1;40(12):2230–42. doi: 10.1038/s41388-021-01689-6 (PMC7994200; doi:10.1038/s41388-021-01689-6)
Supplement: Supplementary file 1 — supplementary figure legends [file 41388_2021_1689_MOESM1_ESM.docx]

**Figure S1. RNA expression of HuR in human cancers and correlates with CMTM6 mRNA levels.** Pearson’s correlation coefficients are shown with *p* values. TPM, transcript per million. ACC, adrenocortical carcinoma; BLCA, urothelial bladder carcinoma; BRCA, breast cancer;

CESC, cervical squamous cell carcinoma; CHOL, cholangiocarcinoma; COAD, colorectal adenocarcinoma; DLBC, diffuse large B-cell lymphoma; GBM, glioblastoma multiforme; HNSC, head and neck squamous cancer; KICH, chromophobe renal cell carcinoma; KIRC, clear cell kidney carcinoma; KIRP, papillary kidney carcinoma; LAML, acute myeloid leukemia; LGG, lower grade glioma; LIHC, liver hepatocellular carcinoma; LUAD, lung adenocarcinoma; MESO, mesothelioma; PAAD, pancreatic ductal adenocarcinoma; PCPG, pheochromocytoma and paraganglioma; PRAD, prostate adenocarcinoma; READ, rectum

adenocarcinoma; SARC, sarcoma; SKCM, cutaneous melanoma; STAD, stomach cancer; TGCT, testicular germ cell cancer; THCA, papillary thyroid carcinoma; THYM, thymoma; UCEC, uterine corpus endometrial carcinoma; UCS, uterine carcinosarcoma; UVM, uveal melanoma.

**Figure S2. HuR up-regulated CMTM6 in Caki-1 and 769-p cells. A.** Establishment HuR-overexpressing and silencing cell lines in Caki-1 and 769-p cells, respectively. Relative expression of HuR was quantified by real-time PCR. Three biological repeats (Mean ± SD). **B.** Quantification of endogenous CMTM6 transcripts in Caki-1 (E.V and HuR-overexpressing) and 769-p (shNT, shHuR-1 and shHuR-2) cells. Three biological repeats (Mean ± SD). **C.** Western blots analysis of HuR and CMTM6 protein in Caki-1 (E.V and HuR-overexpressing) and 769-p (control, shHuR-1 and shHuR-2) cells. ** *p* < 0.01, ****p* < 0.001.

**Figure S3. Relative expression of HuR and CMTM6 mRNA in KIRC.** Tumor=523 (red), normal=100 (black), n.s, no significance.

**Figure S4. CMTM6 mRNA decay in Caki-1 (E.V and HuR-overexpressing, left) and 769-p (shNT, shHuR-1 and shHuR-2, right) cells.** Relative CMTM6 mRNA was quantified at 0, 2, 4 and 6 h post-treatment with 50 mM of α-amanitin. Representative of three independent experiments.

**Figure S5. Overexpression of HuR in 293T.** Validated by western blots analysis (FL, T1, T2 or T3 in combination with either E.V or HuR). Representative result of three repeats.

**Figure S6. Scramble mutations of AREs disrupted binding of HuR to CMTM6 3’UTR-fused luciferase and compromised its stability. A**. RNA-IP analysis of relative enrichment of CMTM6 3’UTR-fused luciferase mRNAs in HuR-immunoprecipitated species in 293T cells. Three biological repeats (Mean ± SD). **B**. CMTM6 3’UTR-fused luciferase mRNA decay in 293T cells at 0, 2, 4 and 6 h post-treatment with 10 μg/ml of Actinomycin D. Three biological repeats. n.s: no significance, * p < 0.05, ** p < 0.01.

**Figure S7. HuR up-regulated cell surface PD-L1 via CMTM6. A**. Western blots analysis of PD-L1 in Caki-1 (E.V and HuR-overexpressing) and 769-p (control, shHuR-1 and shHuR-2) cells without or with exposure to IFN-γ. **B**. Western blots analysis of PD-L1, CMTM6 and HuR protein in Caki-1 (E.V and HuR-overexpressing in combination with either control or shCMTM6) and 769-p (shNT and shHuR-1 in combination with either empty control or HuR-overexpression) cells without or with exposure to IFN-γ. Representative result of three repeats.

**Figure S8. HuR imposed no significant influences on PD-L1 translation efficiency. A**. Representative polysome profiles of 786-0 cells (E.V and HuR). **B.** Distribution of PD-L1 and β-actin mRNA across the fractions of the sucrose gradient in response to HuR overexpression in 786-0 cells. Data are representative of two independent experiments.

**Figure S9. Correlation analysis of HuR and PD-L1 in renal tumors based on IHC intensity scores.** R^2^=0.268, p=0.0194, n=20.

**Figure S10. Co-localization and interaction of PD-L1 with CMTM6 in response to HuR overexpression and knockdown. A**. Immunofluorescence image of cell surface PD-L1 staining with anti-human PD-L1 and CMTM6 antibodies in 786-0 (E.V and HuR, upper) and ACHN (shNT and shHuR, lower) cells. **B**. Western blots determination of CMTM6 in PD-L1 immunoprecipitated complex in response to HuR overexpression in 786-0 cells (upper) and knockdown in ACHN cells (lower).

**Figure S11.** **MS-444 decreased HuR-upregulatedCMTM6 transcript levels.** Real-time PCR analysis of endogenous CMTM6 transcripts in HuR-proficient 786-0 and Caki-1 cells treated with vehicle, 10, 20, 40, 60, 80 and 100 μM of MS-444. Three biological repeats (Mean ± SD). n.s: no significance, * p < 0.05, ** p < 0.01, ***p < 0.001.

**Figure S12. MS-444 showed no influences on HuR expression.** Relative expression of HuR was determined at both transcript and protein levels by real-time PCR and western blots analysis in 769-p and ACHN cells exposed to MS-444 (50 μM). Three biological repeats (Mean ± SD). n.s: no significance.

**Figure S13. MS-444 abolished binding of HuR on CMTM6 3’UTR-fused luciferase.** RNA-IP analysis of relative enrichment of CMTM6 3’UTR-fused luciferase mRNA in HuR-immunoprecipitated complex in 293T cells treated with 50 μM of MS-444. Three biological repeats (Mean ± SD). ** p < 0.01.

**Figure S14. Prolonged half-life of CMTM6 transcripts in HuR-proficient cells was decreased by MS-444 treatment.** CMTM6 mRNA decay in 786-0 (E.V and HuR, left) and Caki-1 (E.V and HuR, right) cells at 0, 2, 4 and 6 h post-treatment with 10 μg/ml of Actinomycin D. Three biological repeats.

**Figure S15. HuR inhibition with CMLD-2 abolished both CMTM6 and PD-L1 up-regulation. A**. CMTM6 mRNA abundance was determined by real-time PCR in both 786-0 and Caki-1 (E.V and HuR-overexpressing) cells with or without CMLD-2 treatments. Three biological repeats (Mean ± SD). **B**. Quantitative PCR analysis of PD-L1 mRNA in 786-0 and Caki-1 (E.V and HuR-overexpressing) cells in response to CMLD-2. Three biological repeats (Mean ± SD). **C**. Western blots analysis of PD-L1, CMTM6 and HuR proteins in 786-0 and Caki-1 (E.V and HuR-overexpressing) cells in presence of CMLD-2. n.s: no significance, ** p < 0.01, ***p < 0.001.

**Figure S16. MS-444 restored IL-2 secretion suppressed by HuR in 786-0 and Caki-1 cells. A.** IL-2 secretion was determined by ELISA in Jurkat co-culture with 786-0 (E.V and HuR-overexpressing in combination with PD-L1 knockdowns) and ACHN (control, shHuR-1 and shHuR-2 in combination with PD-L1 overexpression) cells at 48 and 72 h, respectively. Three independent measurements (Mean ± SD). **B**. IL-2 secretion was determined by ELISA in Jurkat co-culture with 786-0 and Caki-1 (E.V and HuR-overexpressing) cells in absence and presence of MS-444 at 48 and 72 h, respectively. Three independent measurements (Mean ± SD). * p < 0.05, ** p < 0.01, ***p < 0.001, ****p < 0.0001.

**Figure S17. The impacts of HuR-, CMTM6- and PD-L1 knockdown on IL-2 production.** IL-2 production by PD-1^Low^, PD-1^High^ primary human T cells transduced with the human MART-I-specific 1D3 TCR and PD-1, and co-cultured with MART-I peptide pre-loaded ACHN cells (shNT and shHuR, upper), 786-0 cells (shNT and shCMTM6, middle; shNT and shPD-L1, lower).

**Figure S18. Correlation analysis of HuR with CMTM4 mRNA levels in TCGA human cancers.** Pearson’s correlation coefficients are shown with *p* values. TPM, transcript per million. ACC, adrenocortical carcinoma; BLCA, urothelial bladder carcinoma; BRCA, breast cancer;

CESC, cervical squamous cell carcinoma; CHOL, cholangiocarcinoma; COAD, colorectal adenocarcinoma; DLBC, diffuse large B-cell lymphoma; GBM, glioblastoma multiforme; HNSC, head and neck squamous cancer; KICH, chromophobe renal cell carcinoma; KIRC, clear cell kidney carcinoma; KIRP, papillary kidney carcinoma; LAML, acute myeloid leukemia; LGG, lower grade glioma; LIHC, liver hepatocellular carcinoma; LUAD, lung adenocarcinoma; MESO, mesothelioma; PAAD, pancreatic ductal adenocarcinoma; PCPG, pheochromocytoma and paraganglioma; PRAD, prostate adenocarcinoma; READ, rectum

adenocarcinoma; SARC, sarcoma; SKCM, cutaneous melanoma; STAD, stomach cancer; TGCT, testicular germ cell cancer; THCA, papillary thyroid carcinoma; THYM, thymoma; UCEC, uterine corpus endometrial carcinoma; UCS, uterine carcinosarcoma; UVM, uveal melanoma.

**Figure S19. HuR showed no significant regulation on CMTM4 expression. A**. Correlation analysis of △Ct values of endogenous HuR and CMTM4 mRNAs in panel of ccRCC cell lines (786-0, 769-p, A498, A704, ACHN, Caki-1, Caki2, RCC4). **B.** Quantification of endogenous CMTM4 transcripts in 786-0 (E.V and HuR-overexpressing) and ACHN (control, shHuR-1 and shHuR-2) cells. Three biological repeats (Mean ± SD). n.s: no significance.
