# Supplementary figures and images for: HuR up-regulates cell surface PD-L1 via stabilizing CMTM6 transcript in cancer
Source: Oncogene. 2021 Mar 1;40(12):2230–42. doi: 10.1038/s41388-021-01689-6 (PMC7994200; doi:10.1038/s41388-021-01689-6)

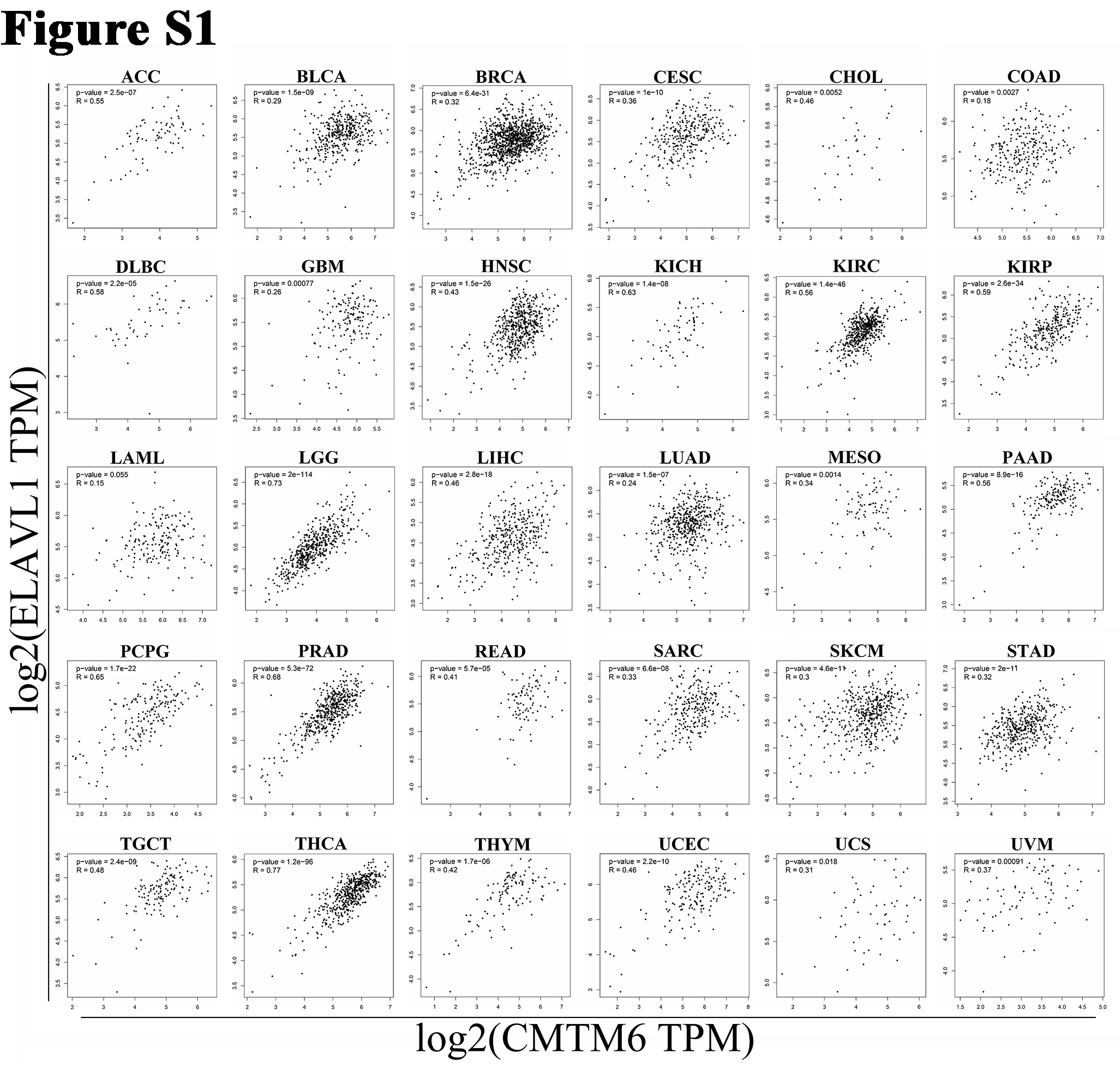

Supplement: Supplementary file 2 — Figure S1. RNA expression of HuR in human cancers and correlates with CMTM6 mRNA levels. [file 41388_2021_1689_MOESM2_ESM.jpg]

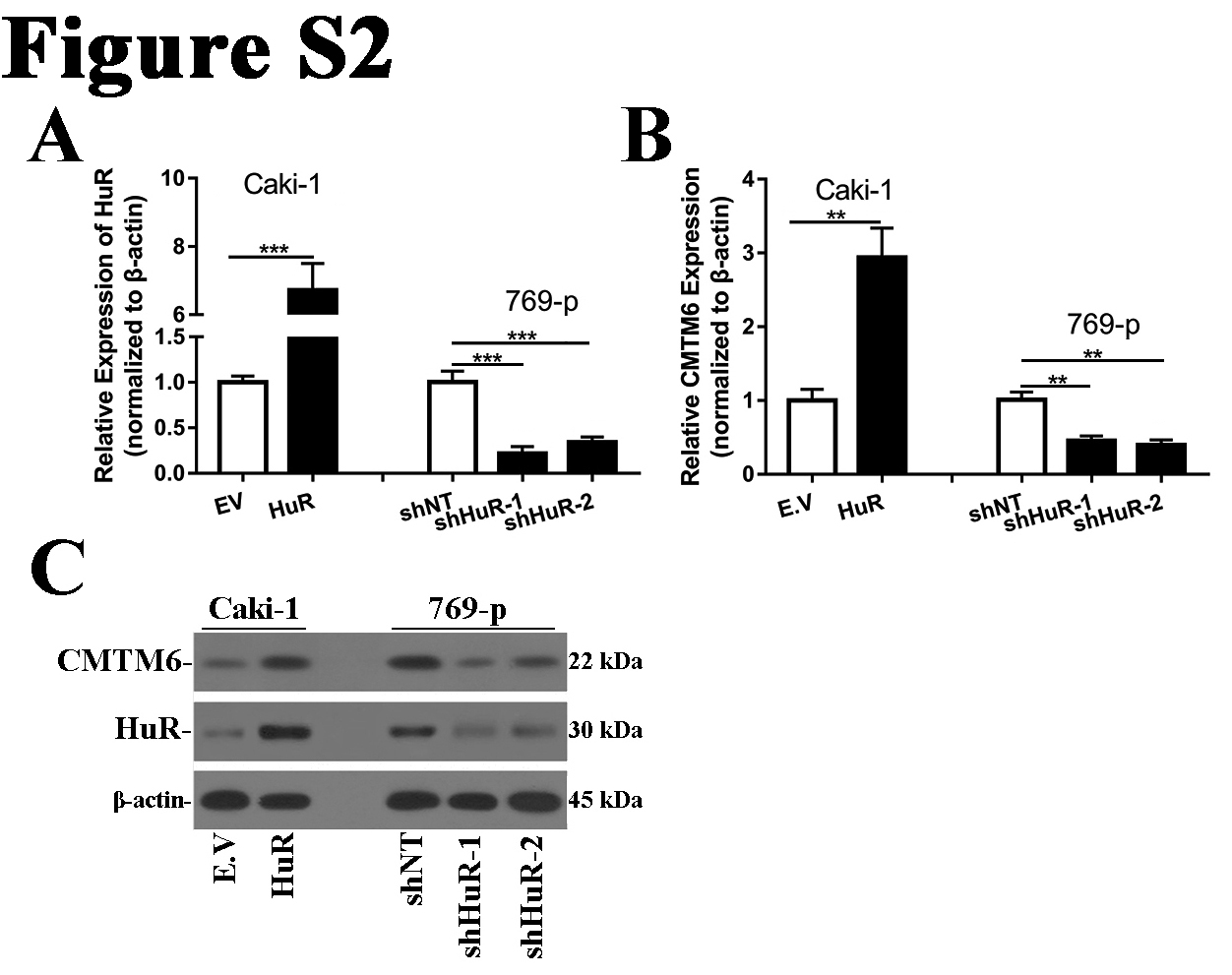

Supplement: Supplementary file 3 — Figure S2. HuR up-regulated CMTM6 in Caki-1 and 769-p cells. [file 41388_2021_1689_MOESM3_ESM.jpg]

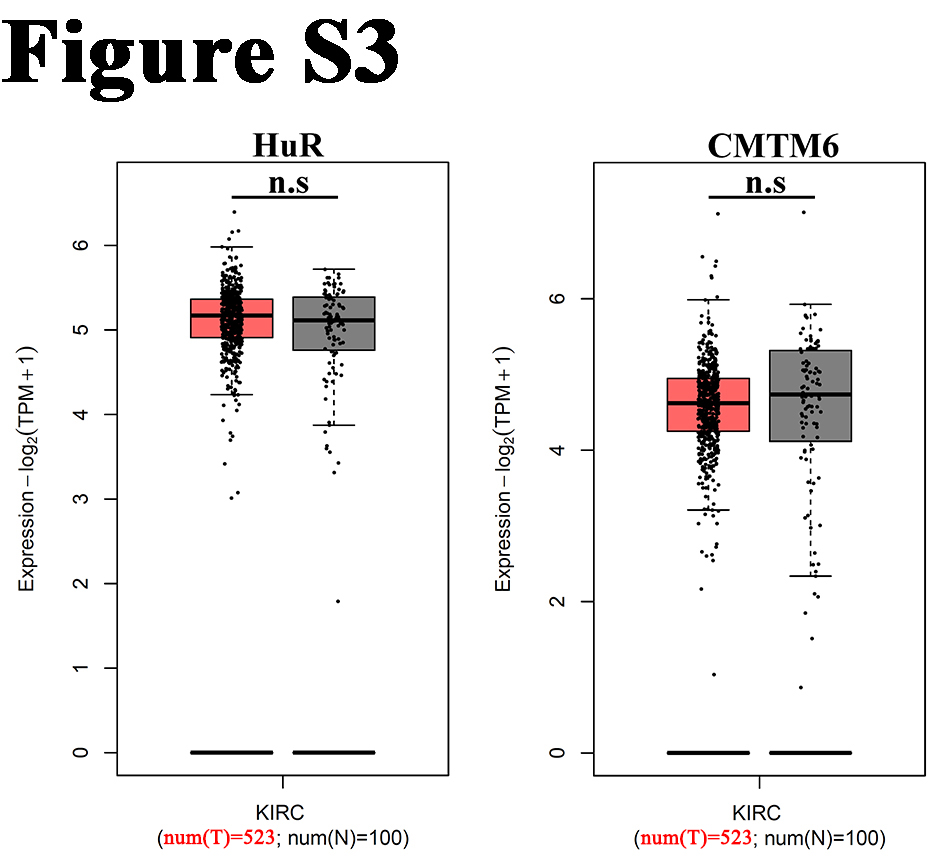

Supplement: Supplementary file 4 — Figure S3. Relative expression of HuR and CMTM6 mRNA in KIRC. [file 41388_2021_1689_MOESM4_ESM.jpg]

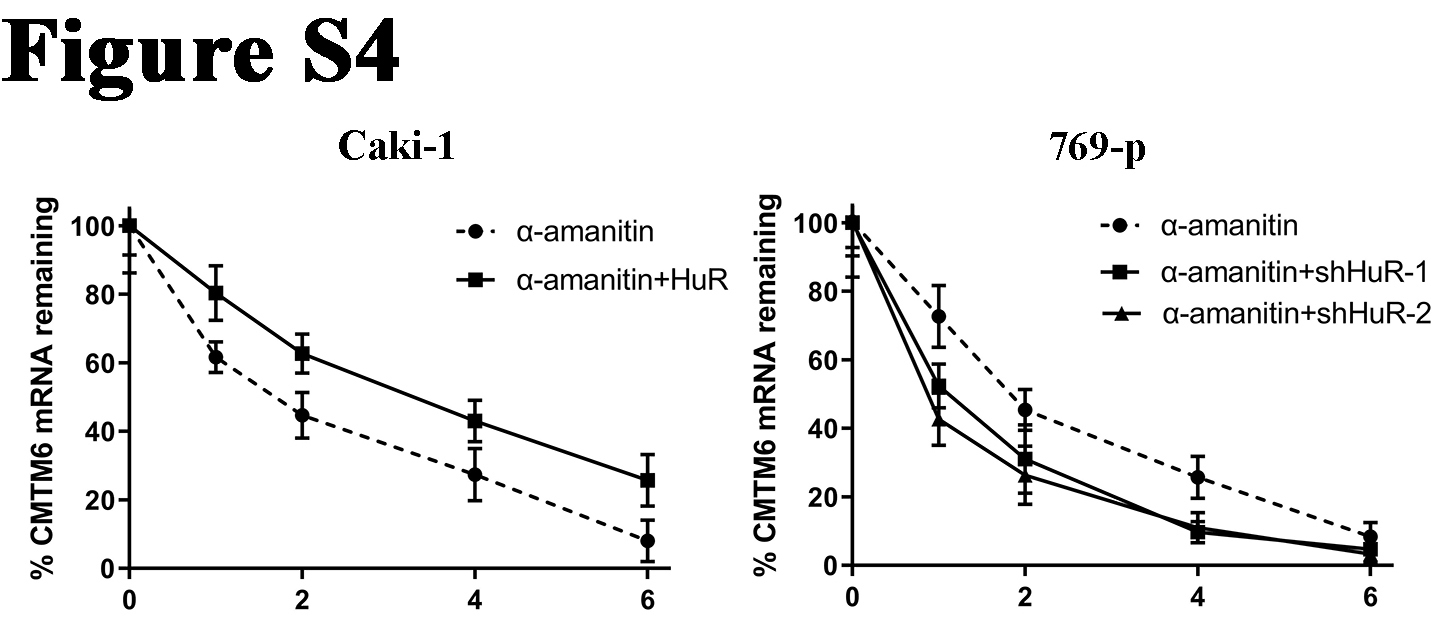

Supplement: Supplementary file 5 — Figure S4. CMTM6 mRNA decay in Caki-1 (E.V and HuR-overexpressing, left) and 769-p (shNT, shHuR-1 and shHuR-2, right) cells. [file 41388_2021_1689_MOESM5_ESM.jpg]

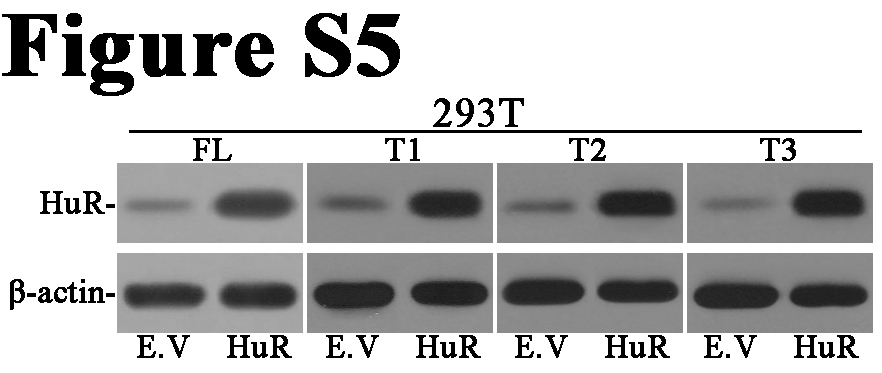

Supplement: Supplementary file 6 — Figure S5. Overexpression of HuR in 293T. [file 41388_2021_1689_MOESM6_ESM.jpg]

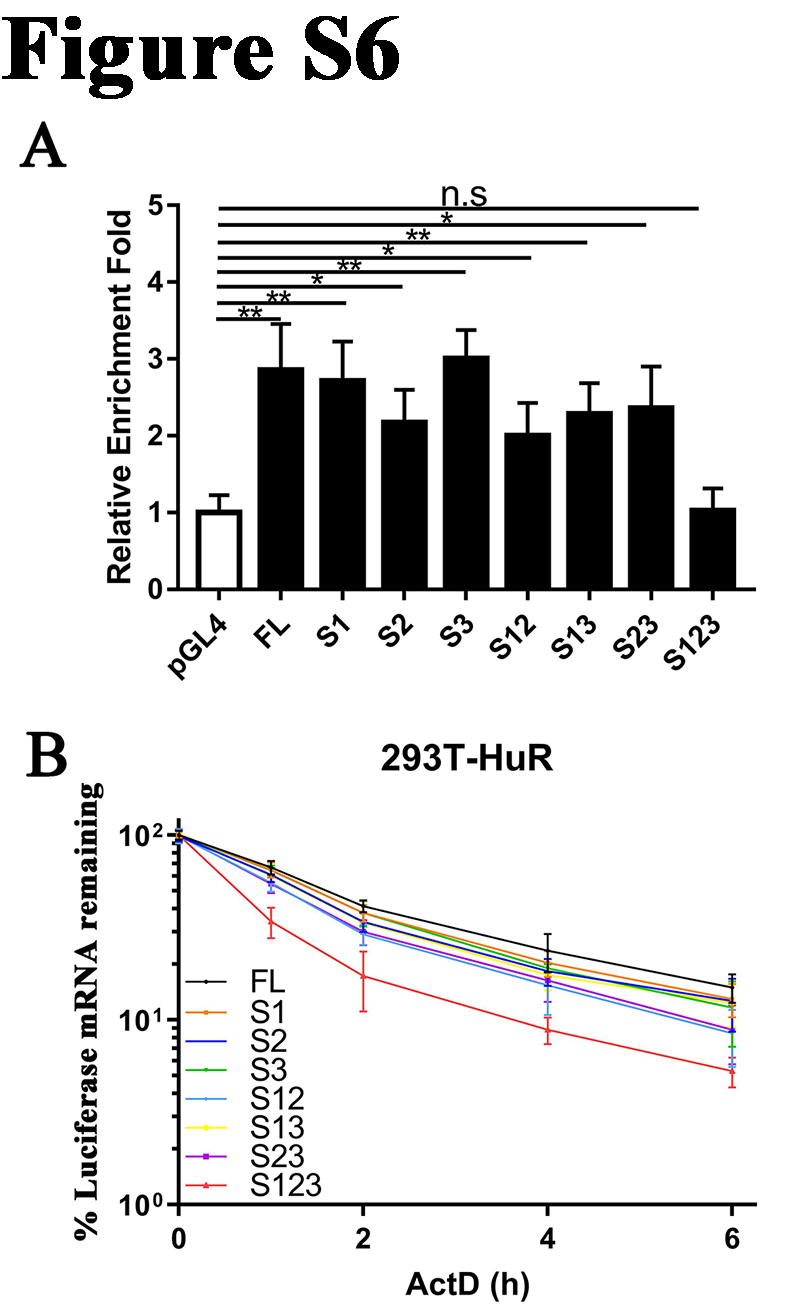

Supplement: Supplementary file 7 — Figure S6. Scramble mutations of AREs disrupted binding of HuR to CMTM6 3’UTR-fused luciferase and compromised its stability. [file 41388_2021_1689_MOESM7_ESM.jpg]

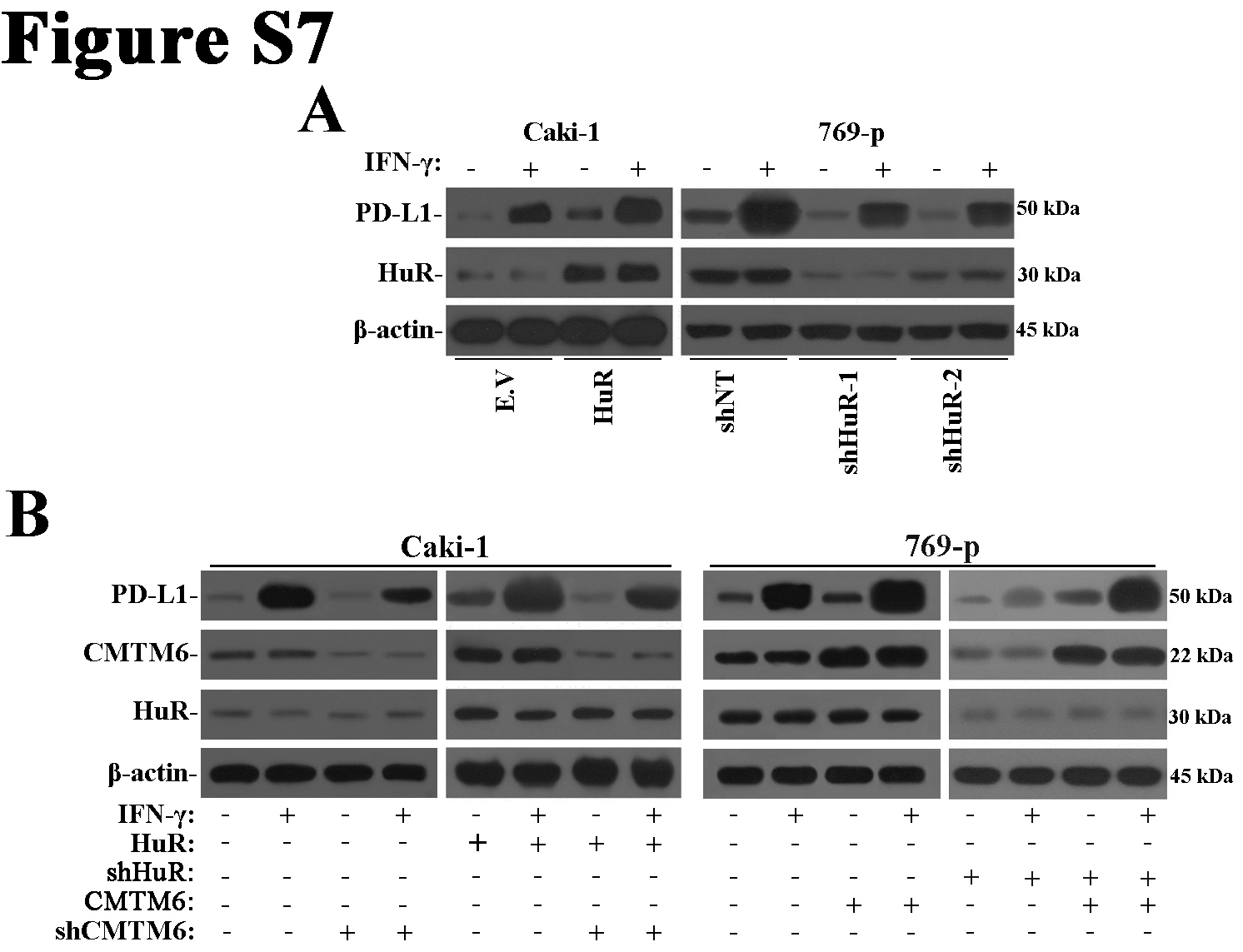

Supplement: Supplementary file 8 — Figure S7. HuR up-regulated cell surface PD-L1 via CMTM6. [file 41388_2021_1689_MOESM8_ESM.jpg]

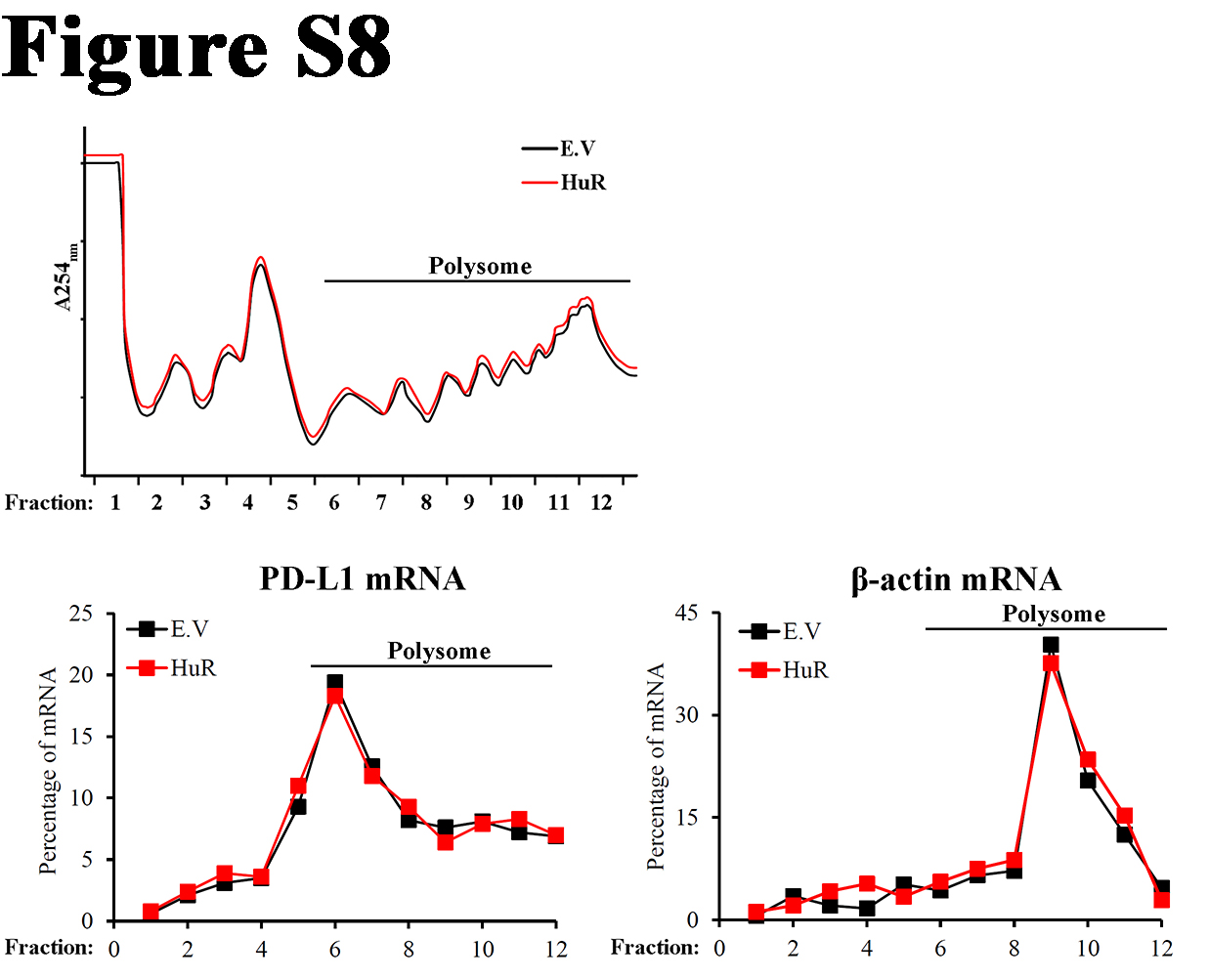

Supplement: Supplementary file 9 — Figure S8. HuR imposed no significant influences on PD-L1 translation efficiency. [file 41388_2021_1689_MOESM9_ESM.jpg]

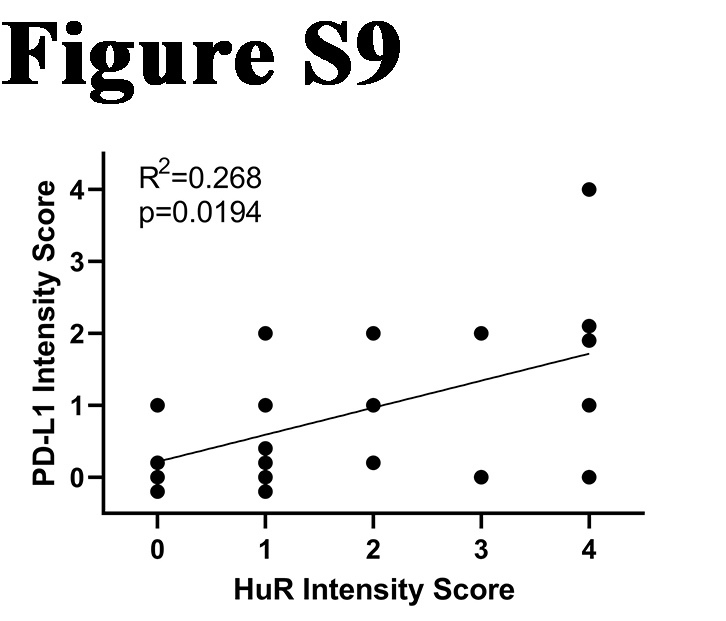

Supplement: Supplementary file 10 — Figure S9. Correlation analysis of HuR and PD-L1 in renal tumors based on IHC intensity scores. [file 41388_2021_1689_MOESM10_ESM.jpg]

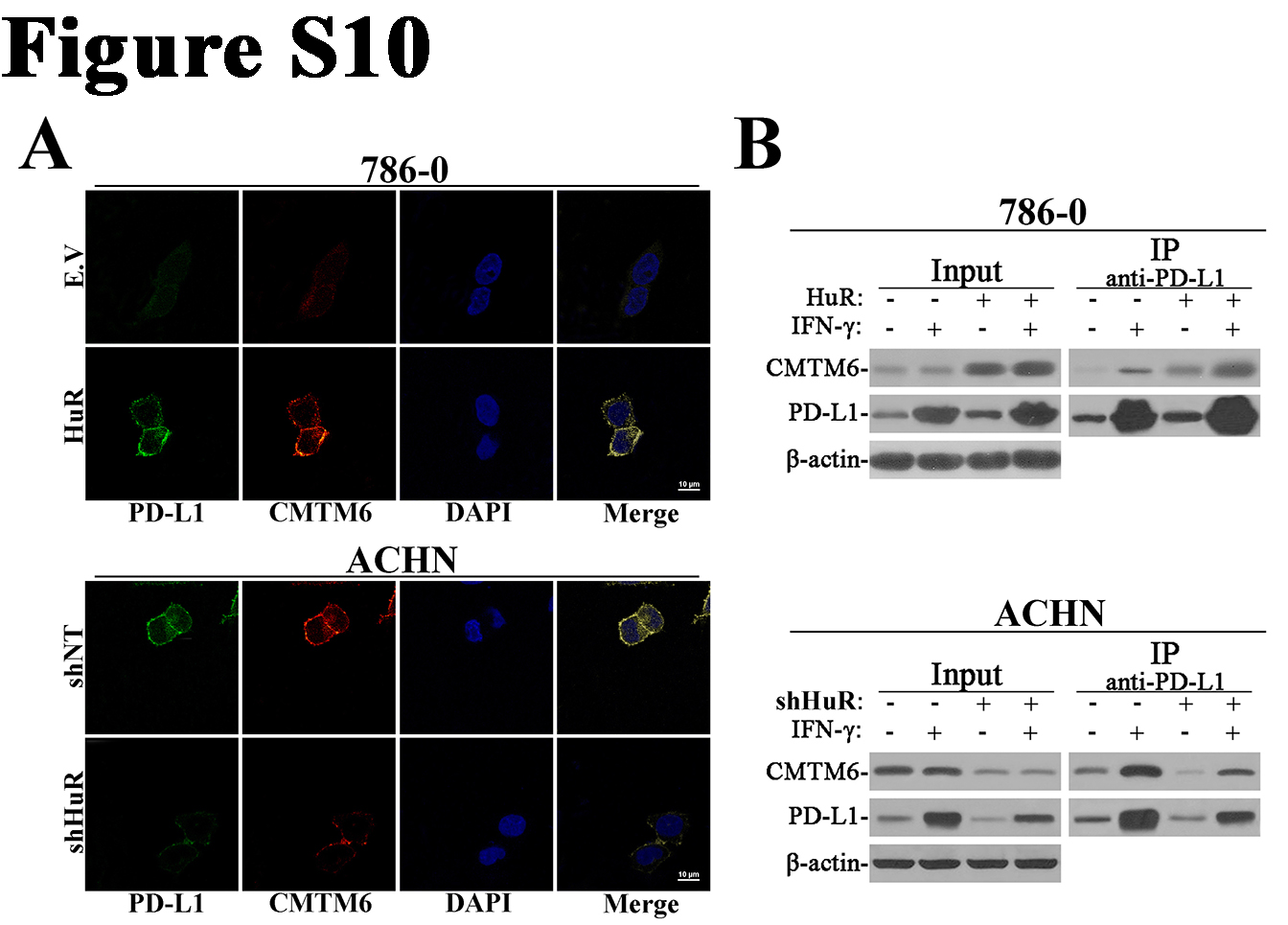

Supplement: Supplementary file 11 — Figure S10. Co-localization and interaction of PD-L1 with CMTM6 in response to HuR overexpression and knockdown. [file 41388_2021_1689_MOESM11_ESM.jpg]

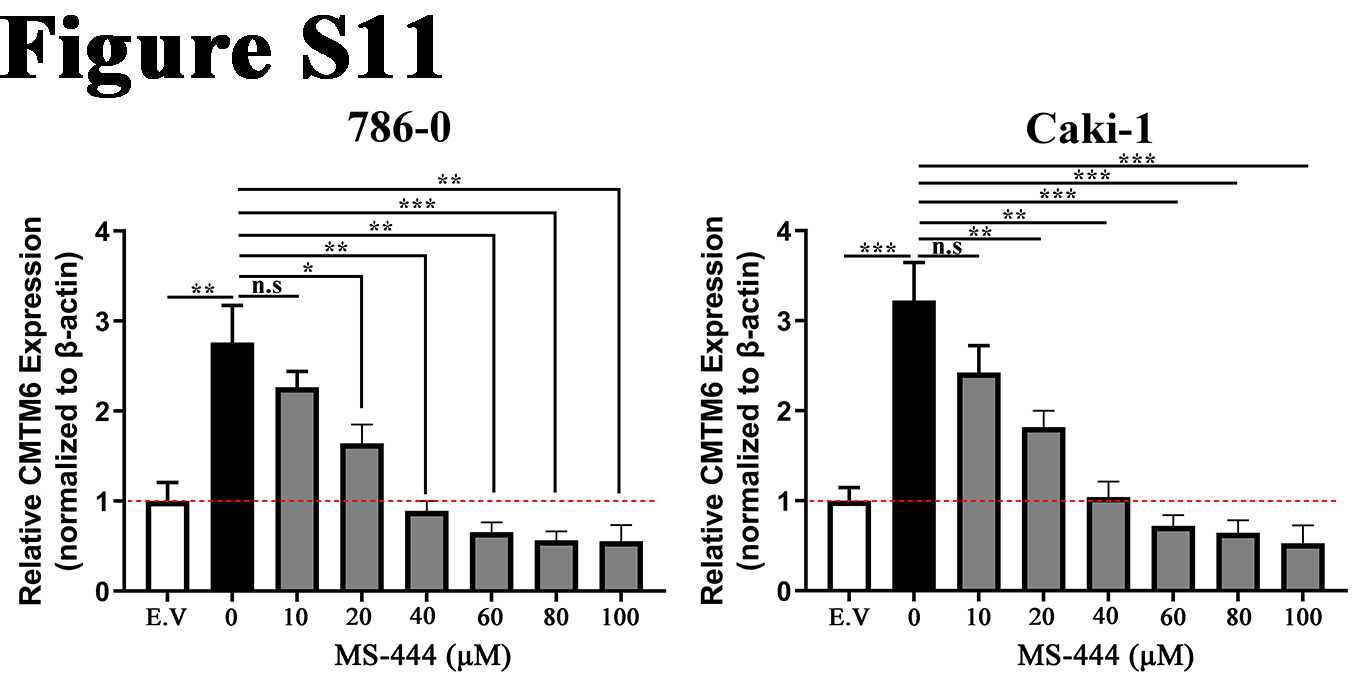

Supplement: Supplementary file 12 — Figure S11. MS-444 decreased HuR-upregulatedCMTM6 transcript levels. [file 41388_2021_1689_MOESM12_ESM.jpg]

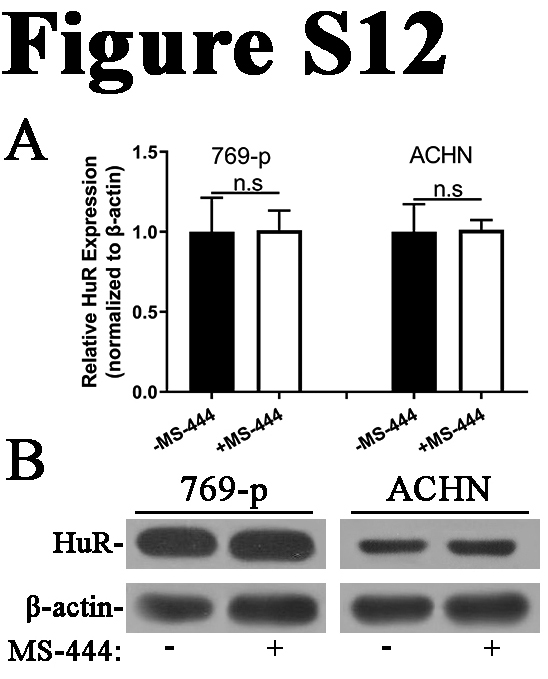

Supplement: Supplementary file 13 — Figure S12. MS-444 showed no influences on HuR expression. [file 41388_2021_1689_MOESM13_ESM.jpg]

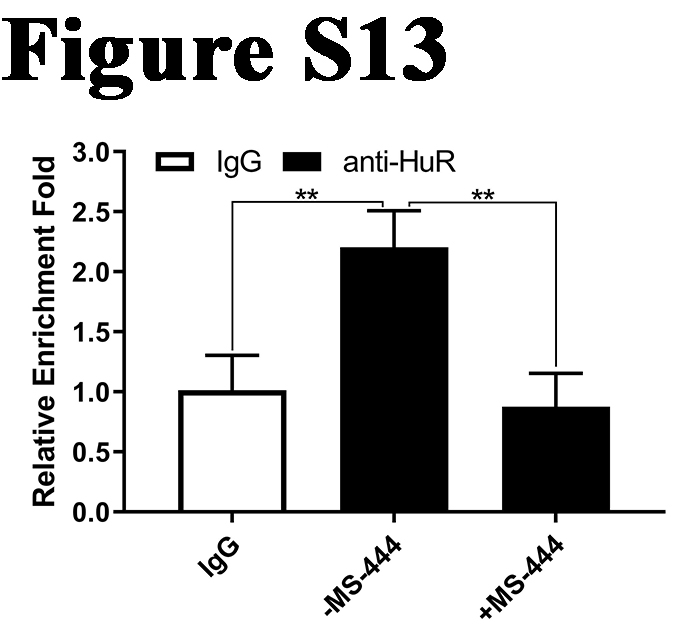

Supplement: Supplementary file 14 — Figure S13. MS-444 abolished binding of HuR on CMTM6 3’UTR-fused luciferase. [file 41388_2021_1689_MOESM14_ESM.jpg]

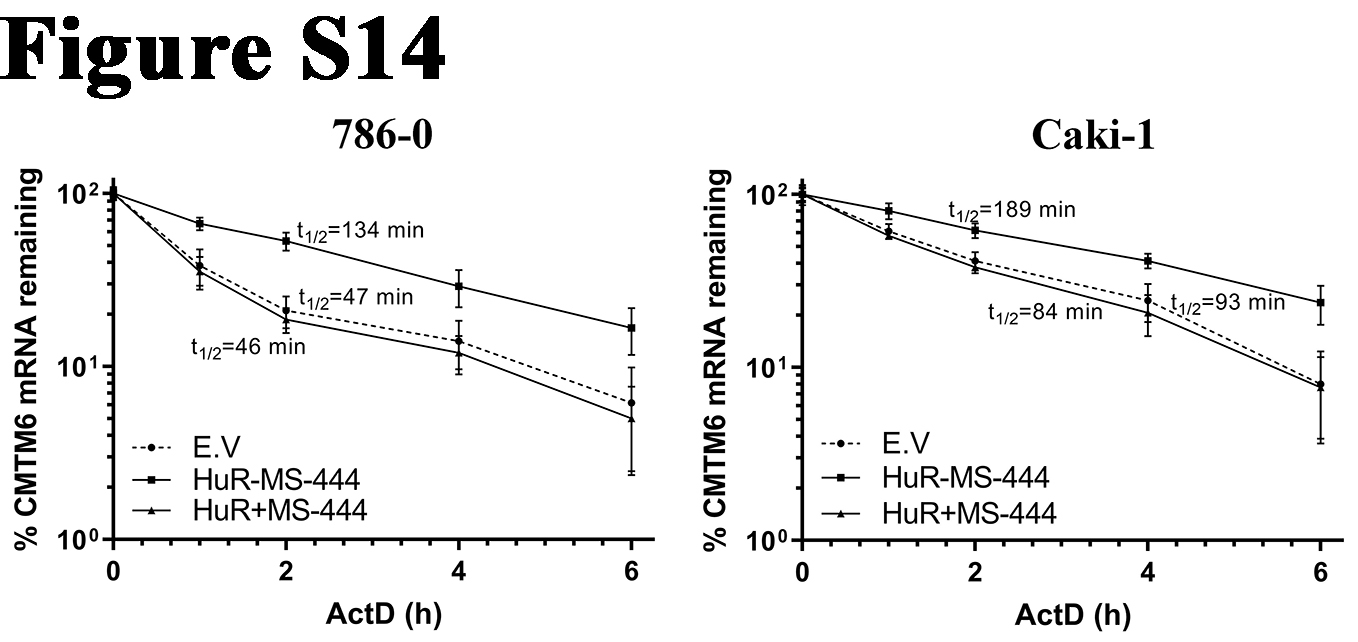

Supplement: Supplementary file 15 — Figure S14. Prolonged half-life of CMTM6 transcripts in HuR-proficient cells was decreased by MS-444 treatment. [file 41388_2021_1689_MOESM15_ESM.jpg]

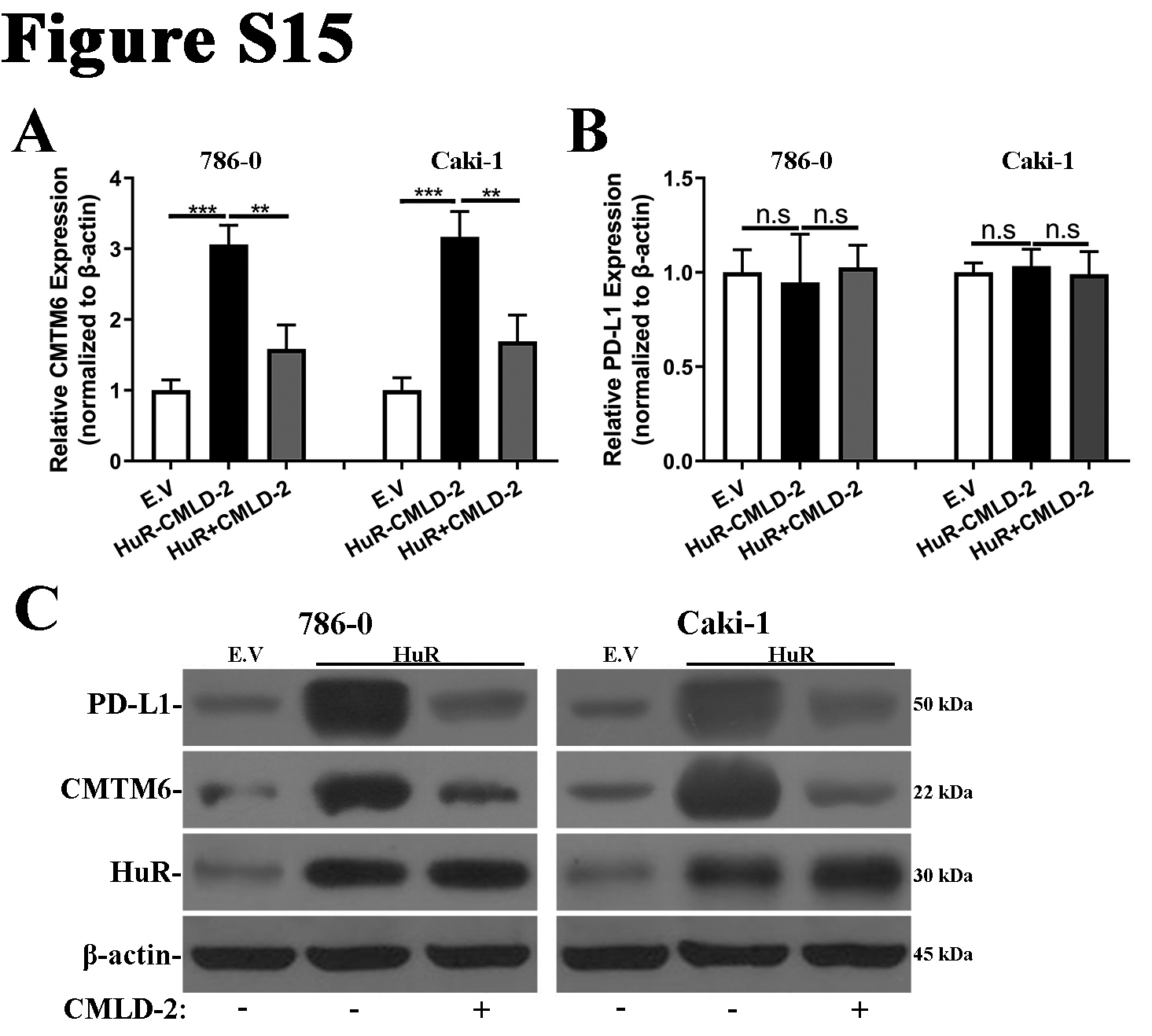

Supplement: Supplementary file 16 — Figure S15. HuR inhibition with CMLD-2 abolished both CMTM6 and PD-L1 up-regulation. [file 41388_2021_1689_MOESM16_ESM.jpg]

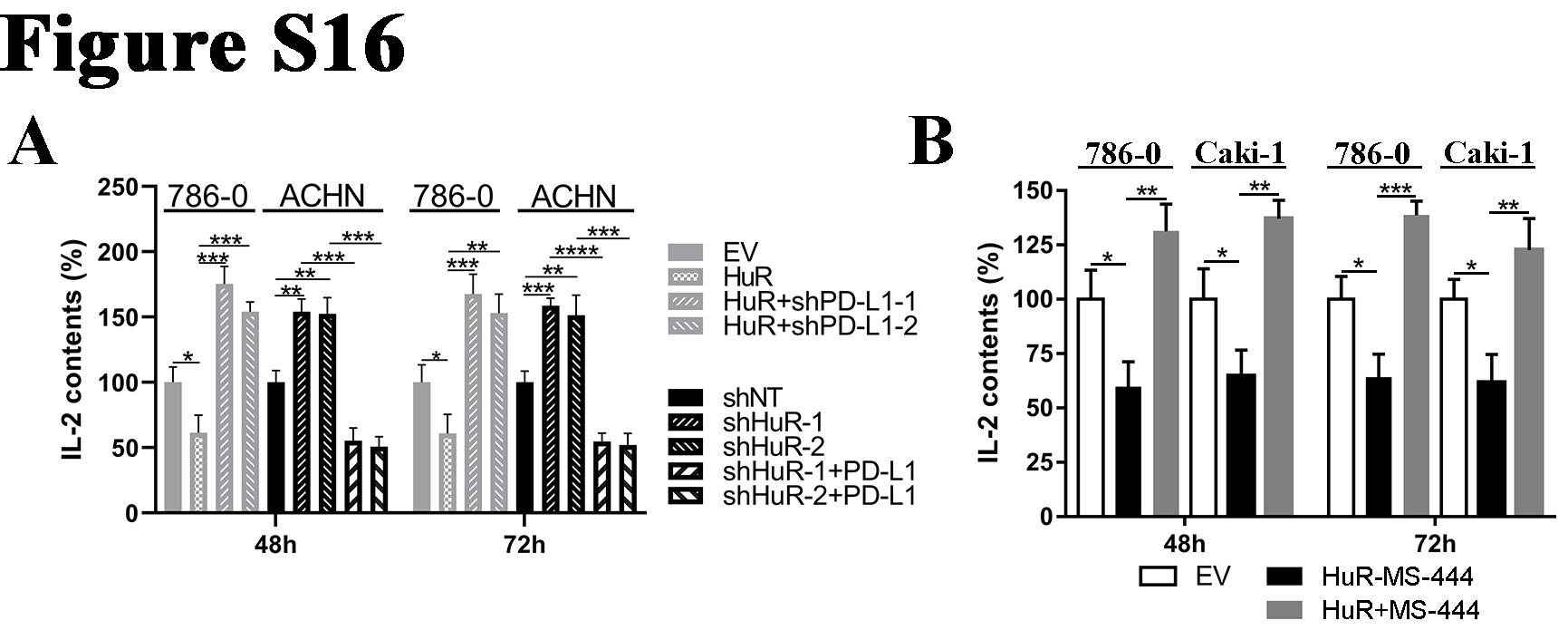

Supplement: Supplementary file 17 — Figure S16. MS-444 restored IL-2 secretion suppressed by HuR in 786–0 and Caki-1 cells. [file 41388_2021_1689_MOESM17_ESM.jpg]

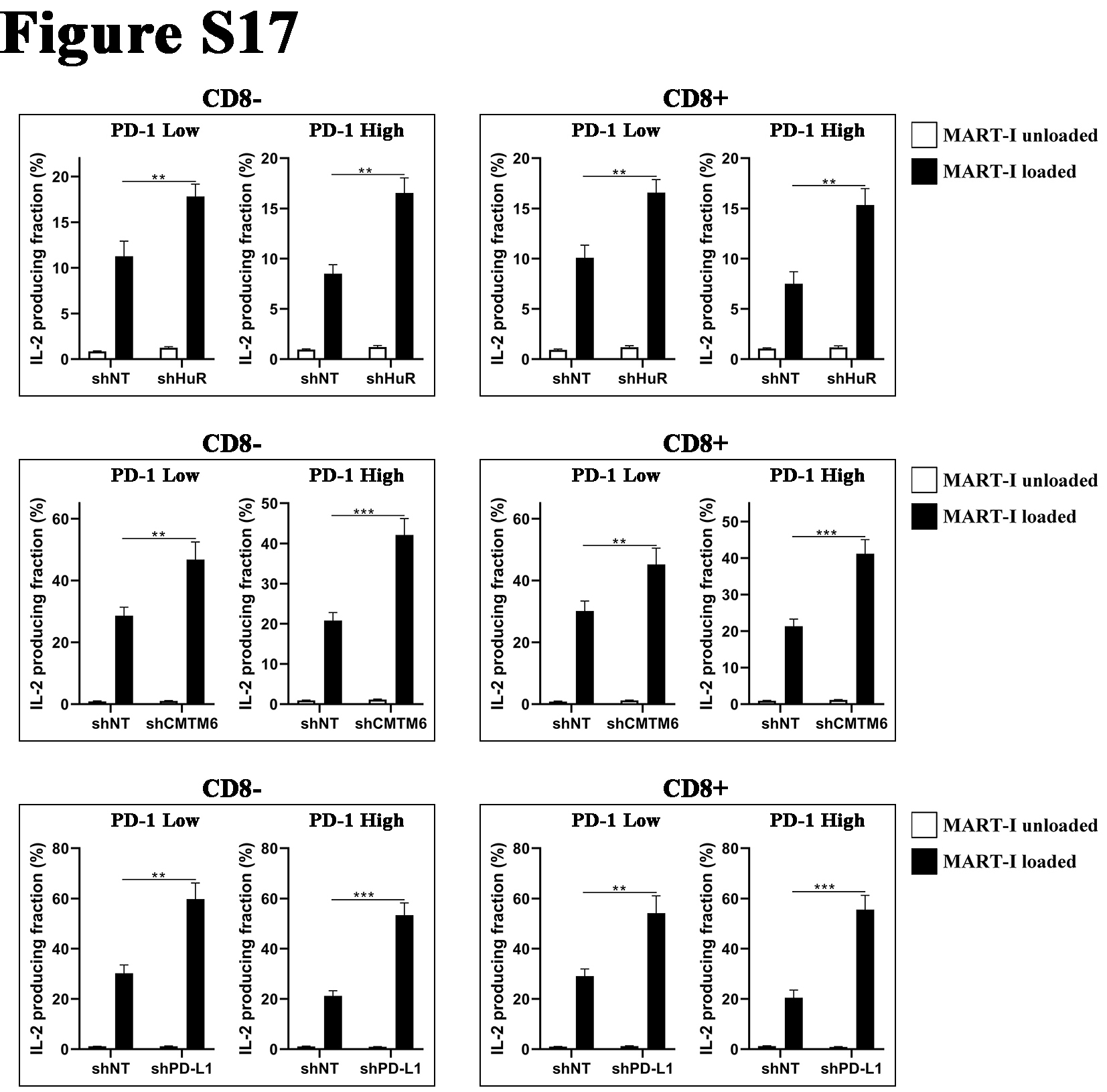

Supplement: Supplementary file 18 — Figure S17. The impacts of HuR-, CMTM6- and PD-L1 knockdown on IL-2 production. [file 41388_2021_1689_MOESM18_ESM.jpg]

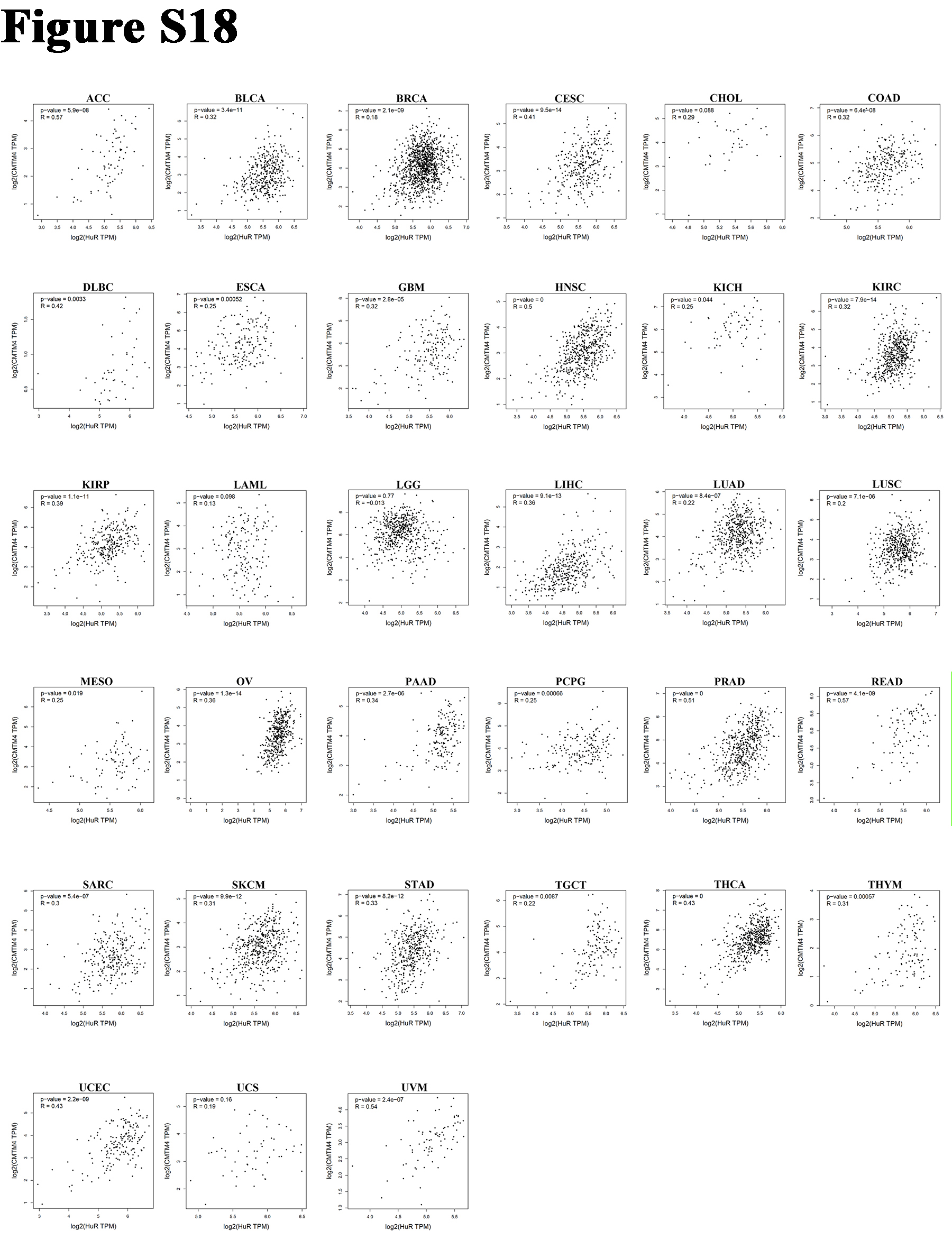

Supplement: Supplementary file 19 — Figure S18. Correlation analysis of HuR with CMTM4 mRNA levels in TCGA human cancers. [file 41388_2021_1689_MOESM19_ESM.jpg]

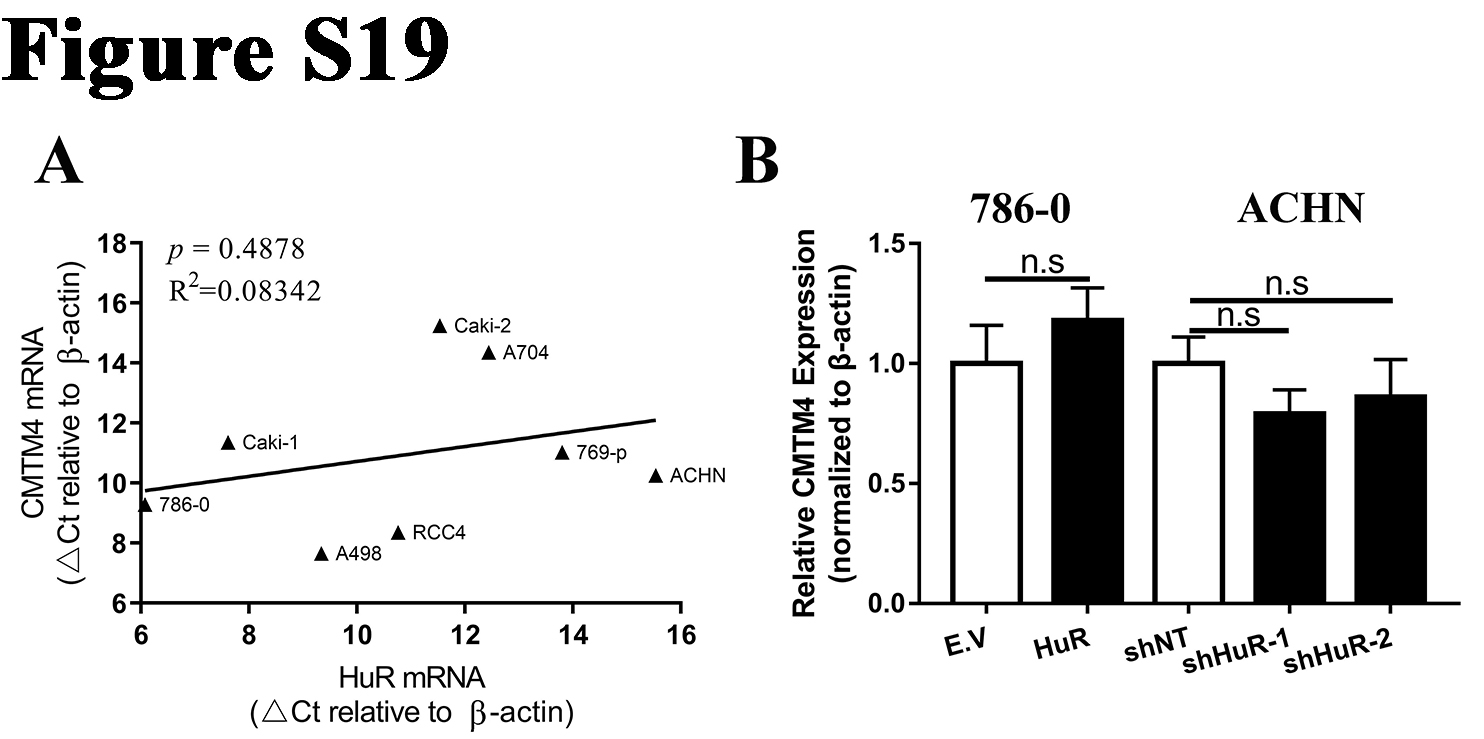

Supplement: Supplementary file 20 — Figure S19. HuR showed no significant regulation on CMTM4 expression. [file 41388_2021_1689_MOESM20_ESM.jpg]
